# Supplementary material for: Which vaccine attributes foster vaccine uptake? A cross-country conjoint experiment
Source: PLoS One. 2022 May 4;17(5):e0266003. doi: 10.1371/journal.pone.0266003 (PMC9067644; doi:10.1371/journal.pone.0266003)
Supplement: S1 File — (ZIP) [file pone.0266003.s001.zip › suppl. information.pdf]

Supplementary material

Table SI1: Sociodemographics France

Variable	N	Sample%	Population%
Age	1801		
18-24	208	11.5%	11%
25-44	610	33.9%	34%
45-54	306	17.0%	17%
55+	677	37.6%	36%
Gender	1798		
Male	855	47.4%	48%
Female	943	52.3%	52%
Region	1704		
Alsace	49	2.7%	3%
Aquitaine	91	5.0%	5%
Auvergne	36	2.0%	2%
Basse-Normandie	47	2.6%	2%
Bourgogne	48	2.7%	3%
Bretagne	95	5.3%	5%
Centre	74	4.1%	4%
Champagne-Ardenne	37	2.1%	2%
Corse	6	0.3%	1%
Franche-Comte	34	1.9%	2%
Haute-Normandie	54	3.0%	3%
Ile-de-France	328	18.2%	18%
Languedoc-Roussillon	75	4.2%	4%
Limousin	20	1.1%	1%
Lorraine	72	4.0%	4%
Midi-Pyrenees	92	5.1 %	5%
Nord-Pas-de-Calais	117	6.5%	6%
Pays de la Loire	102	5.7%	6%
Picardie	54	3.0%	3%
Poitou-Charentes	35	1.9%	3%
Provence-Alpes-Cote d'Azur	149	8.3%	8%
Rhone-Alpes	179	9.9%	10%

Table SI2: Sociodemographics Germany

Variable	N	Sample%	Population%
Age	1799		
18-24	184	10.2%	10%
25-44	489	27.2%	31%
45-54	313	17.4%	18%
55+	813	45.2%	41%
Gender	1793		
Male	891	49.5%	52%
Female	902	50.1%	48%
Region	1814		
Baden-Wurttemberg	237	13.2%	13%
Bayern	270	15.0%	15%
Berlin	86	4.8%	4%
Brandenburg	59	3.3%	3%
Bremen	19	1.1%	1%
Hamburg	54	3.0%	2%
Hessen	134	7.6%	7%
Mecklenburg-Vorpommern	34	1.9%	2%
Niedersachsen	186	9.3%	10%
Nordrhein-Westfalen	391	21.7%	22%
Rheinland-Pfalz	84	4.7%	5%
Saarland	18	1,0%	1%
Sachsen	99	5.5%	5%
Sachsen-Anhalt	44	2.4%	3%
Schleswig-Holstein	57	3.2%	3%
Thuringen	42	2.3%	3%

Table SI3: Sociodemographics Sweden

Variable	N	Sample%	Population%
Age	1829		
18-24	230	12.6%	10%
25-44	559	30.5%	31%
45-54	271	14.8%	18%
55+	769	42.0%	41%
Gender	1825		
Male	907	49.5%	49%
Female	918	50.1%	51%
Region	1752		
Blekinge lan	32	1.8%	2%
Dalarnas lan	46	2.6%	3%
Gavleborgs lan	56	3.2%	3%
Gotlands lan	17	1.0%	1%
Hallands lan	42	2.4%	3%
Jamtlands lan	23	1.3%	1%
Jonkopings lan	53	3.0%	4%
Kalmar lan	57	2.1%	3.2%
Kronobergs lan	35	2.0%	2%
Norrbottens lan	35	2.0%	3%
Orebro lan	58	3.3%	3%
Ostergotlands lan	86	4.2%	5%
Skane lan	242	13.8%	13%
Sodermanlands lan	57	3.2%	3%
Stockholms lan	406	23.1%	22%
Uppsalas lan	71	4,0 %	4%
Varmlands lan	33	1.9%	3%
Vasterbottens lan	44	2.5%	3%
Vasternorrlands lan	47	2.7%	3%
Vastmanlands lan	43	2.4%	3%
Vastra Gotalands lan	269	15.3%	17%

Figure SI1: AMCEs for likelihood of uptake

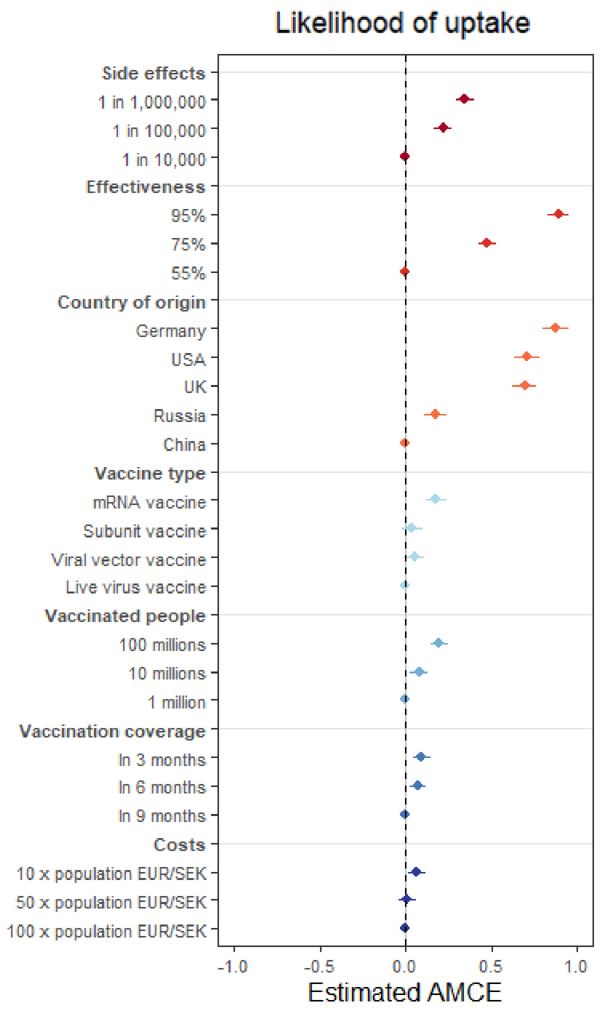

Note. Each dot and error bar represents an AMCE (and its 95% CI). They represent the estimated effect sizes for the attribute levels (compared to the reference attribute level) on likelihood of uptake. Reference categories for AMCEs: Side effects = '1 in 10,000', effectiveness = 55%, country of origin = China, vaccine type = live virus vaccine, vaccinated people = 1 million, vaccination coverage = in 9 months, costs = 100 x population EUR/SEK.

Table SI4: MMs for self-reported likelihood of uptake

	Attribute	Level	Estimate	SE	Lower CI	Upper CI
1	Side effects	1 in 10,000	4.94	0.04	4.87	5.01
2	Side effects	1 in 100,000	5.16	0.03	5.09	5.23
3	Side effects	1 in 1,000,000	5.30	0.04	5.22	5.37
4	Effectiveness	55%	4.68	0.04	4.61	4.75
5	Effectiveness	75%	5.15	0.04	5.08	5.22
6	Effectiveness	95%	5.57	0.04	5.50	5.64
7	Country	China	4.64	0.04	4.56	4.72
8	Country	Russia	4.82	0.04	4.74	4.90
9	Country	Great Britain	5.33	0.04	5.25	5.41
10	Country	USA	5.36	0.04	5.28	5.43
11	Country	Germany	5.52	0.04	5.44	5.60
12	Type	Live Virus Vaccine	5.07	0.04	4.99	5.14
13	Type	Viral Vector Vaccine	5.11	0.04	5.04	5.18
14	Type	Subunit Vaccine	5.11	0.04	5.04	5.18
15	Type	mRNA Vaccine	5.25	0.04	5.17	5.32
16	People	1 million	5.04	0.04	4.97	5.11
17	People	10 million	5.12	0.04	5.05	5.19
18	People	100 million	5.23	0.04	5.17	5.30
19	Duration	In 9 months	5.08	0.04	5.01	5.15
20	Duration	In 6 months	5.16	0.04	5.09	5.22
21	Duration	In 3 months	5.17	0.04	5.10	5.24
22	Cost	100 x population EUR/SEK	5.11	0.04	5.04	5.18
23	Cost	50 x population EUR/SEK	5.12	0.04	5.05	5.19
24	Cost	10 x population EUR/SEK	5.17	0.04	5.10	5.24

Table SI5: AMCEs for self-reported likelihood of uptake

	Attribute	Level	Estimate	SE	Lower CI	Upper CI
1	Side effects	1 in 10,000	0.00			
2	Side effects	1 in 100,000	0.22	0.03	0.17	0.27
3	Side effects	1 in 1,000,000	0.35	0.03	0.29	0.40
4	Effectiveness	55%	0.00			
5	Effectiveness	75%	0.48	0.03	0.42	0.53
6	Effectiveness	95%	0.89	0.03	0.83	0.95
7	Country	China	0.00			
8	Country	Russia	0.17	0.03	0.11	0.24
9	Country	Great Britain	0.69	0.04	0.62	0.77
10	Country	USA	0.71	0.04	0.64	0.78
11	Country	Germany	0.88	0.04	0.80	0.95
12	Type	Live Virus Vaccine	0.00			
13	Type	Viral Vector Vaccine	0.05	0.03	-0.01	0.11
14	Type	Subunit Vaccine	0.04	0.03	-0.02	0.10
15	Type	mRNA Vaccine	0.18	0.03	0.12	0.24
16	People	1 million	0.00			
17	People	10 million	0.08	0.03	0.03	0.13
18	People	100 million	0.20	0.03	0.15	0.25
19	Duration	In 9 months	0.00			
20	Duration	In 6 months	0.07	0.03	0.02	0.12
21	Duration	In 3 months	0.09	0.03	0.04	0.14
22	Cost	100 x population EUR/SEK	0.00			
23	Cost	50 x population EUR/SEK	0.01	0.03	-0.04	0.06
24	Cost	10 x population EUR/SEK	0.07	0.03	0.02	0.12

Note: Reference categories for AMCEs: Side effects = '1 in 10,000', effectiveness = 55%, country of origin = China, vaccine type = live virus vaccine, vaccinated people = 1 million, vaccination coverage = in 9 months, costs = 100 x population EUR/SEK.

Figure SI2: MMs for vaccine choice

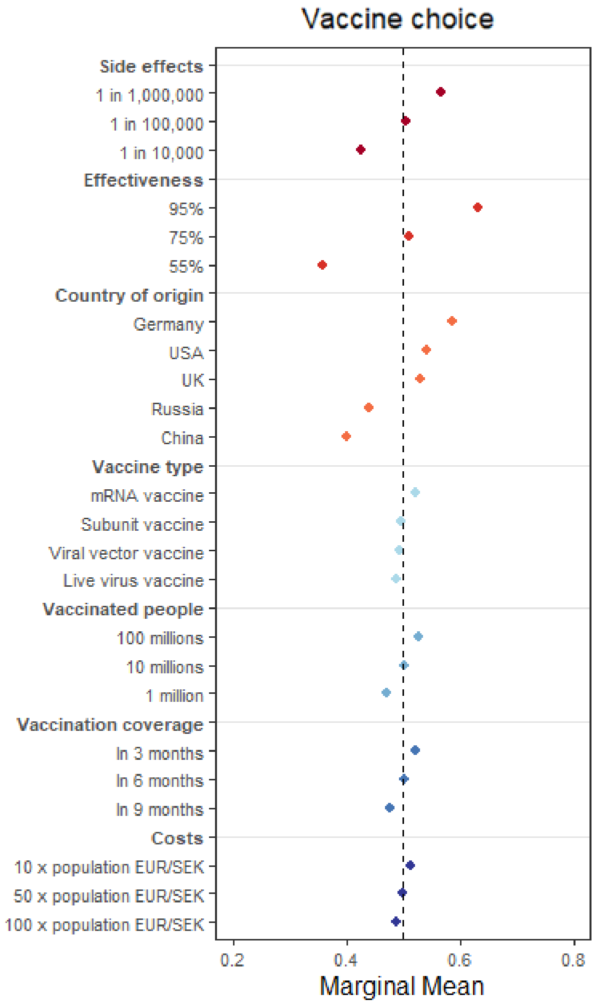

Note. Each dot and error bar represents the MM (and its 95% CI) for vaccine choice.

Figure SI3: AMCEs for vaccine choice

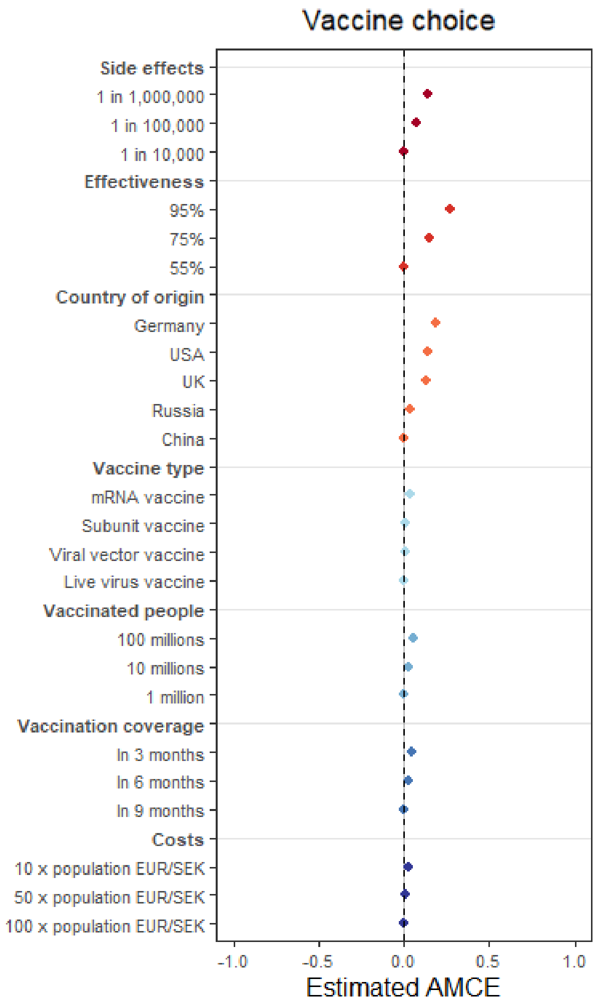

Note. Each dot and error bar represents an AMCE (and its 95% CI). They represent the estimated effect sizes for the attribute levels (compared to the reference attribute level) on vaccine choice. Reference categories for AMCEs: Side effects = '1 in 10,000', effectiveness = 55%, country of origin = China, vaccine type = live virus vaccine, vaccinated people = 1 million, vaccination coverage = in 9 months, costs = 100 x population EUR/SEK.

Table SI6: MMs for vaccine choice

	Attribute	Level	Estimate	SE	Lower CI	Upper CI
1	Side effects	1 in 10,000	0.43	0.00	0.42	0.43
2	Side effects	1 in 100,000	0.50	0.00	0.50	0.51
3	Side effects	1 in 1,000,000	0.57	0.00	0.56	0.57
4	Effectiveness	55%	0.36	0.00	0.35	0.36
5	Effectiveness	75%	0.51	0.00	0.51	0.51
6	Effectiveness	95%	0.63	0.00	0.63	0.64
7	Country	China	0.40	0.00	0.39	0.41
8	Country	Russia	0.44	0.00	0.43	0.45
9	Country	Great Britain	0.53	0.00	0.52	0.54
10	Country	USA	0.54	0.00	0.53	0.55
11	Country	Germany	0.59	0.00	0.58	0.60
12	Type	Live Virus Vaccine	0.49	0.00	0.48	0.49
13	Type	Viral Vector Vaccine	0.49	0.00	0.49	0.50
14	Type	Subunit Vaccine	0.50	0.00	0.49	0.50
15	Type	mRNA Vaccine	0.52	0.00	0.52	0.53
16	People	1 million	0.47	0.00	0.47	0.48
17	People	10 million	0.50	0.00	0.50	0.51
18	People	100 million	0.53	0.00	0.52	0.53
19	Duration	In 9 months	0.48	0.00	0.47	0.48
20	Duration	In 6 months	0.50	0.00	0.50	0.51
21	Duration	In 3 months	0.52	0.00	0.52	0.53
22	Cost	100 x population EUR/SEK	0.49	0.00	0.48	0.49
23	Cost	50 x population EUR/SEK	0.50	0.00	0.50	0.50
24	Cost	10 x population EUR/SEK	0.51	0.00	0.51	0.52

Table SI7: AMCEs for vaccine choice

	Attribute	Level	Estimate	SE	Lower CI	Upper CI
1	Side effects	1 in 10,000	0.00			
2	Side effects	1 in 100,000	0.08	0.00	0.07	0.08
3	Side effects	1 in 1,000,000	0.14	0.00	0.13	0.15
4	Effectiveness	55%	0.00			
5	Effectiveness	75%	0.15	0.00	0.14	0.16
6	Effectiveness	95%	0.27	0.00	0.26	0.28
7	Country	China	0.00			
8	Country	Russia	0.04	0.01	0.03	0.05
9	Country	Great Britain	0.13	0.01	0.12	0.14
10	Country	USA	0.14	0.01	0.13	0.15
11	Country	Germany	0.19	0.01	0.17	0.20
12	Type	Live Virus Vaccine	0.00			
13	Type	Viral Vector Vaccine	0.01	0.00	-0.00	0.02
14	Type	Subunit Vaccine	0.01	0.00	-0.00	0.02
15	Type	mRNA Vaccine	0.03	0.01	0.02	0.04
16	People	1 million	0.00			
17	People	10 million	0.03	0.00	0.02	0.04
18	People	100 million	0.06	0.00	0.05	0.07
19	Duration	In 9 months	0.00			
20	Duration	In 6 months	0.02	0.00	0.02	0.03
21	Duration	In 3 months	0.05	0.00	0.04	0.05
22	Cost	100 x population EUR/SEK	0.00			
23	Cost	50 x population EUR/SEK	0.01	0.00	0.00	0.02
24	Cost	10 x population EUR/SEK	0.02	0.00	0.02	0.03

Note: Reference categories for AMCEs: Side effects = '1 in 10,000', effectiveness = 55%, country of origin = China, vaccine type = live virus vaccine, vaccinated people = 1 million, vaccination coverage = in 9 months, costs = 100 x population EUR/SEK.

Figure SI4: MMs and AMCEs for self-reported likelihood of uptake and vaccine choice (respondents ≥ 2 attention checks)

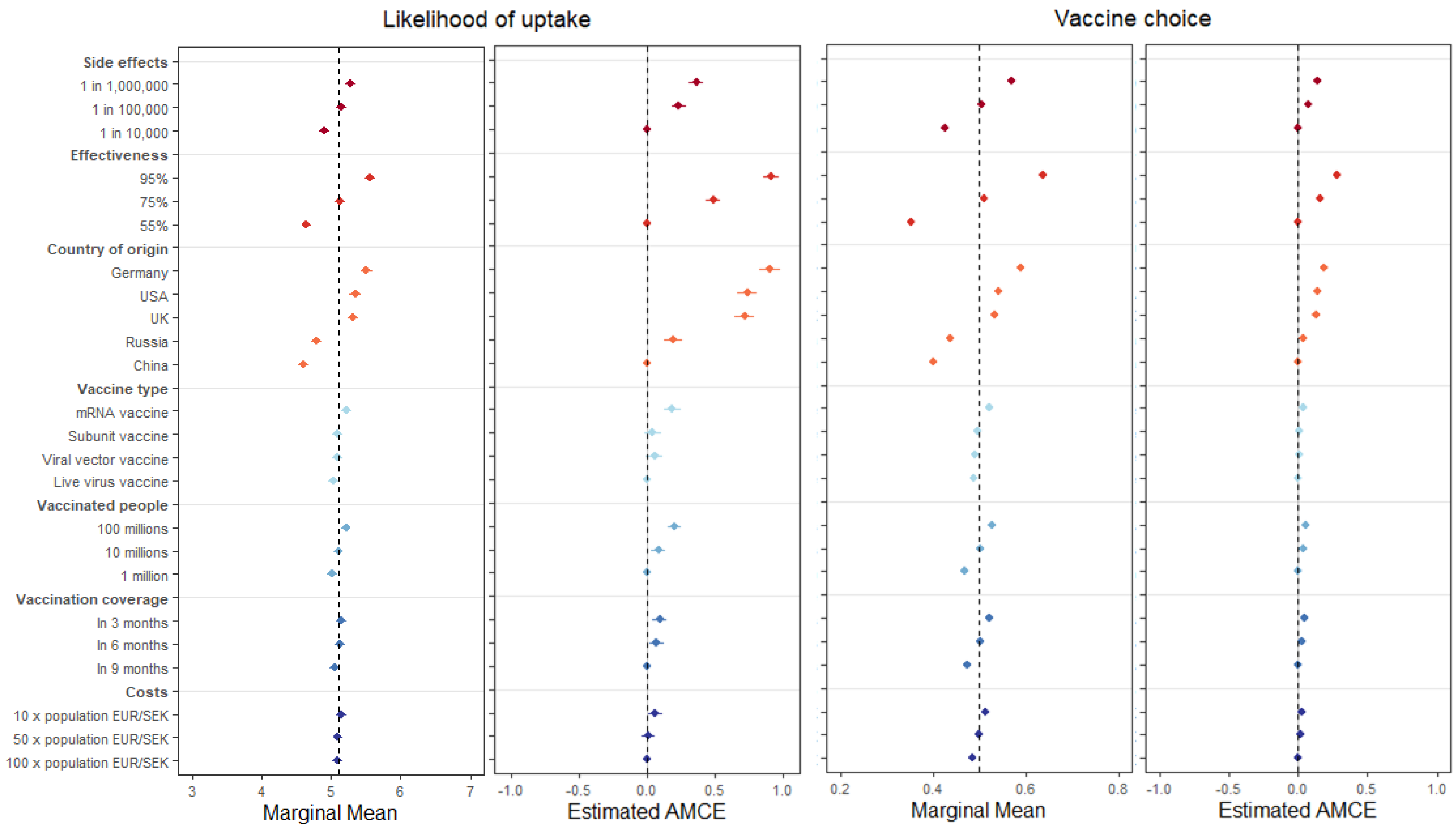

Note. The sample with respondents that passed at least two attention checks (of a total number of four attention checks) was N = 4,739 (France: 1,588; Germany: 1,581; Sweden: 1,570). MMs and AMCEs of vaccine attributes on self-reported likelihoods of uptake (left), and vaccine choice (right). The point estimates are plotted with 95% confidence intervals. The dashed lines for MMs represent the grand mean. Reference categories for AMCEs: Side effects = '1 in 10,000', effectiveness = 55%, country of origin = China, vaccine type = live virus vaccine, vaccinated people = 1 million, vaccination coverage = in 9 months, costs = 100 x population EUR/SEK.

Table SI8: MMs for likelihood of uptake (respondents ≥ 2 attention checks)

	Attribute	Level	Estimate	SE	Lower CI	Upper CI
1	Side effects	1 in 10,000	4.78	0.04	4.71	4.86
2	Side effects	1 in 100,000	5.04	0.04	4.97	5.12
3	Side effects	1 in 1,000,000	5.19	0.04	5.12	5.27
4	Effectiveness	55%	4.50	0.04	4.43	4.58
5	Effectiveness	75%	5.03	0.04	4.96	5.11
6	Effectiveness	95%	5.49	0.04	5.41	5.57
7	Country	China	4.45	0.04	4.37	4.54
8	Country	Russia	4.66	0.04	4.57	4.74
9	Country	Great Britain	5.22	0.04	5.14	5.30
10	Country	USA	5.26	0.04	5.18	5.34
11	Country	Germany	5.45	0.04	5.37	5.53
12	Type	Live Virus Vaccine	4.92	0.04	4.85	5.00
13	Type	Viral Vector Vaccine	4.98	0.04	4.90	5.06
14	Type	Subunit Vaccine	4.99	0.04	4.91	5.07
15	Type	mRNA Vaccine	5.13	0.04	5.05	5.21
16	People	1 million	4.91	0.04	4.83	4.98
17	People	10 million	5.00	0.04	4.92	5.07
18	People	100 million	5.12	0.04	5.04	5.19
19	Duration	In 9 months	4.95	0.04	4.87	5.02
20	Duration	In 6 months	5.03	0.04	4.95	5.10
21	Duration	In 3 months	5.05	0.04	4.97	5.12
22	Cost	100 x population EUR/SEK	4.99	0.04	4.91	5.06
23	Cost	50 x population EUR/SEK	4.99	0.04	4.92	5.07
24	Cost	10 x population EUR/SEK	5.05	0.04	4.97	5.12

Table SI9: AMCEs for likelihood of uptake (respondents $\geq$ 2 attention checks)

	Attribute	Level	Estimate	SE	Lower CI	Upper CI
1	Side effects	1 in 10,000	0.00			
2	Side effects	1 in 100,000	0.26	0.03	0.20	0.31
3	Side effects	1 in 1,000,000	0.40	0.03	0.34	0.46
4	Effectiveness	55%	0.00			
5	Effectiveness	75%	0.53	0.03	0.47	0.59
6	Effectivenessness	95%	0.98	0.03	0.92	1.04
7	Country	China	0.00			
8	Country	Russia	0.20	0.04	0.13	0.27
9	Country	Great Britain	0.78	0.04	0.70	0.86
10	Country	USA	0.80	0.04	0.72	0.88
11	Country	Germany	1.00	0.04	0.91	1.08
12	Type	Live Virus Vaccine	0.00			
13	Type	Viral Vector Vaccine	0.06	0.03	-0.00	0.13
14	Type	Subunit Vaccine	0.07	0.03	0.00	0.13
15	Type	mRNA Vaccine	0.21	0.03	0.14	0.27
16	People	1 million	0.00			
17	People	10 million	0.09	0.03	0.04	0.15
18	People	100 million	0.21	0.03	0.16	0.27
19	Duration	In 9 months	0.00			
20	Duration	In 6 months	0.07	0.03	0.02	0.13
21	Duration	In 3 months	0.10	0.03	0.04	0.16
22	Cost	100 x population EUR/SEK	0.00			
23	Cost	50 x population EUR/SEK	0.01	0.03	-0.05	0.06
24	Cost	10 x population EUR/SEK	0.06	0.03	0.01	0.11

Table SI10: MMs for vaccine choice (respondents $\geq$ 2 attention checks)

	Attribute	Level	Estimate	SE	Lower CI	Upper CI
1	Side effects	1 in 10,000	0.42	0.00	0.41	0.42
2	Side effects	1 in 100,000	0.51	0.00	0.50	0.51
3	Side effects	1 in 1,000,000	0.57	0.00	0.57	0.58
4	Effectiveness	55%	0.34	0.00	0.34	0.35
5	Effectiveness	75%	0.51	0.00	0.50	0.51
6	Effectiveness	95%	0.65	0.00	0.64	0.65
7	Country	China	0.39	0.00	0.38	0.40
8	Country	Russia	0.43	0.00	0.43	0.44
9	Country	Great Britain	0.53	0.00	0.53	0.54
10	Country	USA	0.55	0.00	0.54	0.55
11	Country	Germany	0.60	0.00	0.59	0.60
12	Type	Live Virus Vaccine	0.49	0.00	0.48	0.49
13	Type	Viral Vector Vaccine	0.49	0.00	0.49	0.50
14	Type	Subunit Vaccine	0.50	0.00	0.49	0.50
15	Type	mRNA Vaccine	0.52	0.00	0.52	0.53
16	People	1 million	0.47	0.00	0.46	0.47
17	People	10 million	0.50	0.00	0.50	0.51
18	People	100 million	0.53	0.00	0.52	0.53
19	Duration	In 9 months	0.47	0.00	0.47	0.48
20	Duration	In 6 months	0.50	0.00	0.50	0.51
21	Duration	In 3 months	0.52	0.00	0.52	0.53
22	Cost	100 x population EUR/SEK	0.49	0.00	0.48	0.49
23	Cost	50 x population EUR/SEK	0.50	0.00	0.49	0.50
24	Cost	10 x population EUR/SEK	0.51	0.00	0.51	0.52

Table SI11: AMCEs for vaccine choice (respondents $\geq$ 2 attention checks)

	Attribute	Level	Estimate	SE	Lower CI	Upper CI
1	Side effects	1 in 10,000	0.00			
2	Side effects	1 in 100,000	0.09	0.00	0.08	0.10
3	Side effects	1 in 1,000,000	0.15	0.01	0.14	0.16
4	Effectiveness	55%	0.00			
5	Effectiveness	75%	0.17	0.00	0.16	0.18
6	Effectiveness	95%	0.30	0.01	0.29	0.31
7	Country	China	0.00			
8	Country	Russia	0.04	0.01	0.03	0.05
9	Country	Great Britain	0.14	0.01	0.13	0.16
10	Country	USA	0.15	0.01	0.14	0.16
11	Country	Germany	0.20	0.01	0.19	0.21
12	Type	Live Virus Vaccine	0.00			
13	Type	Viral Vector Vaccine	0.00	0.01	-0.01	0.01
14	Type	Subunit Vaccine	0.01	0.01	-0.00	0.02
15	Type	mRNA Vaccine	0.03	0.01	0.02	0.04
16	People	1 million	0.00			
17	People	10 million	0.04	0.00	0.03	0.04
18	People	100 million	0.06	0.00	0.05	0.07
19	Duration	In 9 months	0.00			
20	Duration	In 6 months	0.03	0.00	0.02	0.04
21	Duration	In 3 months	0.05	0.00	0.04	0.06
22	Cost	100 x population EUR/SEK	0.00			
23	Cost	50 x population EUR/SEK	0.01	0.00	0.00	0.02
24	Cost	10 x population EUR/SEK	0.03	0.00	0.02	0.04

Figure SI5: Subgroup analysis: Cross-country differences

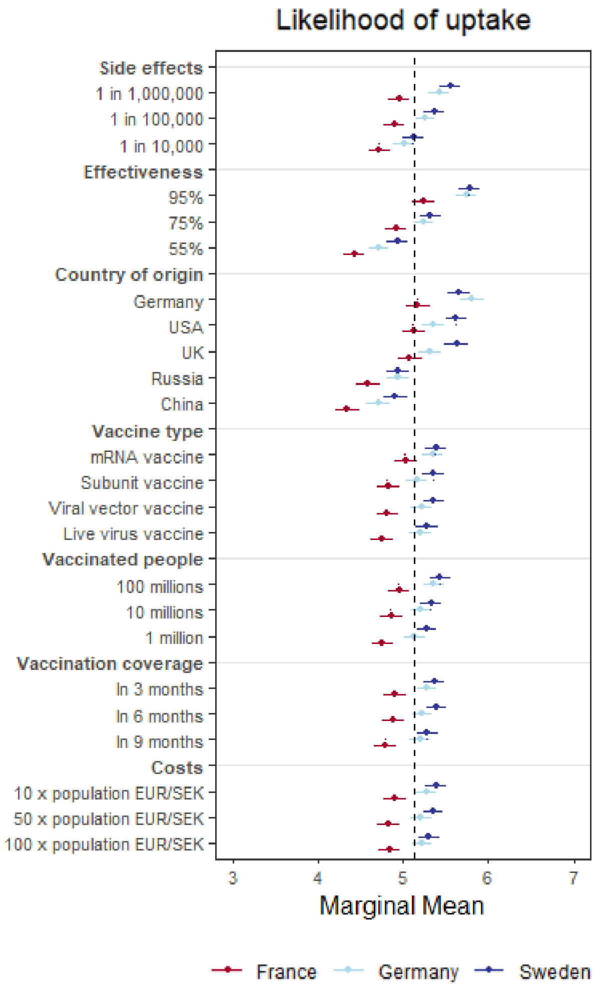

Note. MMs of vaccine attributes on vaccine self-reported likelihood of uptake. The dashed line represents the grand mean. Independent of the vaccine attributes, French respondents report lower likelihoods of uptake than German and Swedish respondents.

Table SI12: Cross-country MMs for self-reported likelihood of uptake

	Country	Attribute	Level	Estimate	SE	Lower CI	Upper CI
1	France	Side effects	1 in 10,000	4.70	0.06	4.57	4.82
2	France	Side effects	1 in 100,000	4.87	0.06	4.75	4.99
3	France	Side effects	1 in 1,000,000	4.92	0.06	4.80	5.05
4	France	Effectiveness	55%	4.40	0.06	4.27	4.52
5	France	Effectiveness	75%	4.90	0.06	4.77	5.02
6	France	Effectiveness	95%	5.21	0.07	5.08	5.34
7	France	Country	China	4.31	0.07	4.17	4.45
8	France	Country	Russia	4.57	0.07	4.43	4.70
9	France	Country	Great Britain	5.05	0.07	4.91	5.19
10	France	Country	USA	5.10	0.07	4.97	5.24
11	France	Country	Germany	5.13	0.07	4.99	5.27
12	France	Type	Live Virus Vaccine	4.73	0.07	4.60	4.86
13	France	Type	Viral Vector Vaccine	4.78	0.07	4.66	4.91
14	France	Type	Subunit Vaccine	4.80	0.07	4.67	4.93
15	France	Type	mRNA Vaccine	5.01	0.07	4.88	5.14
16	France	People	1 million	4.73	0.06	4.61	4.85
17	France	People	10 million	4.83	0.06	4.71	4.96
18	France	People	100 million	4.93	0.06	4.80	5.05
19	France	Duration	In 9 months	4.76	0.06	4.64	4.89
20	France	Duration	In 6 months	4.85	0.06	4.73	4.98
21	France	Duration	In 3 months	4.87	0.06	4.75	5.00
22	France	Cost	100 x population EUR/SEK/HUF	4.80	0.06	4.68	4.93
23	France	Cost	50 x population EUR/SEK/HUF	4.80	0.06	4.68	4.93
24	France	Cost	10 x population EUR/SEK/HUF	4.88	0.06	4.76	5.01
25	Germany	Side effects	1 in 10,000	5.00	0.06	4.88	5.12
26	Germany	Side effects	1 in 100,000	5.26	0.06	5.14	5.37
27	Germany	Side effects	1 in 1,000,000	5.42	0.06	5.30	5.54
28	Germany	Effectiveness	55%	4.71	0.06	4.59	4.83
29	Germany	Effectiveness	75%	5.24	0.06	5.13	5.36
30	Germany	Effectiveness	95%	5.73	0.06	5.61	5.85
31	Germany	Country	China	4.71	0.07	4.57	4.85
32	Germany	Country	Russia	4.94	0.07	4.81	5.08
33	Germany	Country	Great Britain	5.32	0.06	5.19	5.44
34	Germany	Country	USA	5.36	0.07	5.23	5.49
35	Germany	Country	Germany	5.79	0.07	5.66	5.92
36	Germany	Type	Live Virus Vaccine	5.20	0.06	5.08	5.32
37	Germany	Type	Viral Vector Vaccine	5.20	0.06	5.08	5.33
38	Germany	Type	Subunit Vaccine	5.16	0.06	5.04	5.28
39	Germany	Type	mRNA Vaccine	5.34	0.06	5.22	5.47
40	Germany	People	1 million	5.13	0.06	5.02	5.25
41	Germany	People	10 million	5.20	0.06	5.08	5.32
42	Germany	People	100 million	5.35	0.06	5.24	5.46
43	Germany	Duration	In 9 months	5.19	0.06	5.08	5.31
44	Germany	Duration	In 6 months	5.22	0.06	5.10	5.34
45	Germany	Duration	In 3 months	5.27	0.06	5.16	5.39
46	Germany	Cost	100 x population EUR/SEK/HUF	5.23	0.06	5.11	5.34
47	Germany	Cost	50 x population EUR/SEK/HUF	5.20	0.06	5.08	5.31
48	Germany	Cost	10 x population EUR/SEK/HUF	5.26	0.06	5.14	5.38
49	Sweden	Side effects	1 in 10,000	5.12	0.06	5.00	5.24
50	Sweden	Side effects	1 in 100,000	5.36	0.06	5.24	5.48
51	Sweden	Side effects	1 in 1,000,000	5.54	0.06	5.42	5.66
52	Sweden	Effectiveness	55%	4.93	0.06	4.81	5.05
53	Sweden	Effectiveness	75%	5.31	0.06	5.19	5.43
54	Sweden	Effectiveness	95%	5.78	0.06	5.66	5.90
55	Sweden	Country	China	4.91	0.07	4.77	5.04
56	Sweden	Country	Russia	4.94	0.07	4.81	5.08
57	Sweden	Country	Great Britain	5.62	0.07	5.48	5.75
58	Sweden	Country	USA	5.61	0.07	5.49	5.74
59	Sweden	Country	Germany	5.64	0.07	5.51	5.77
60	Sweden	Type	Live Virus Vaccine	5.27	0.07	5.14	5.40
61	Sweden	Type	Viral Vector Vaccine	5.35	0.06	5.23	5.48
62	Sweden	Type	Subunit Vaccine	5.35	0.06	5.23	5.48
63	Sweden	Type	mRNA Vaccine	5.38	0.06	5.26	5.51
64	Sweden	People	1 million	5.27	0.06	5.15	5.39
65	Sweden	People	10 million	5.32	0.06	5.20	5.44
66	Sweden	People	100 million	5.43	0.06	5.31	5.55
67	Sweden	Duration	In 9 months	5.27	0.06	5.15	5.40
68	Sweden	Duration	In 6 months	5.38	0.06	5.27	5.50
69	Sweden	Duration	In 3 months	5.36	0.06	5.24	5.48
70	Sweden	Cost	100 x population EUR/SEK/HUF	5.30	0.06	5.18	5.42
71	Sweden	Cost	50 x population EUR/SEK/HUF	5.35	0.06	5.23	5.47
72	Sweden	Cost	10 x population EUR/SEK/HUF	5.37	0.06	5.25	5.49

Table SI13: Cross-country AMCEs for self-reported likelihood of uptake

	Country	Attribute	Level	Estimate	SE	Lower CI	Upper CI
1	France	Side effects	1 in 10,000	0.00			
2	France	Side effects	1 in 100,000	0.16	0.04	0.07	0.25
3	France	Side effects	1 in 1,000,000	0.21	0.05	0.12	0.30
4	France	Effectiveness	55%	0.00			
5	France	Effectiveness	75%	0.50	0.05	0.41	0.59
6	France	Effectiveness	95%	0.81	0.05	0.71	0.90
7	France	Country	China	0.00			
8	France	Country	Russia	0.25	0.06	0.14	0.37
9	France	Country	Great Britain	0.75	0.06	0.63	0.88
10	France	Country	USA	0.79	0.07	0.66	0.92
11	France	Country	Germany	0.82	0.07	0.69	0.95
12	France	Type	Live Virus Vaccine	0.00			
13	France	Type	Viral Vector Vaccine	0.05	0.05	-0.05	0.15
14	France	Type	Subunit Vaccine	0.07	0.05	-0.03	0.16
15	France	Type	mRNA Vaccine	0.27	0.05	0.16	0.38
16	France	People	1 million	0.00			
17	France	People	10 million	0.11	0.04	0.02	0.20
18	France	People	100 million	0.20	0.05	0.11	0.29
19	France	duration	In 9 months	0.00			
20	France	duration	In 6 months	0.09	0.04	0.00	0.17
21	France	duration	In 3 months	0.12	0.05	0.03	0.21
22	France	cost	100 x population EUR/SEK/HUF	0.00			
23	France	cost	50 x population EUR/SEK/HUF	-0.00	0.04	-0.09	0.08
24	France	cost	10 x population EUR/SEK/HUF	0.09	0.04	-0.00	0.17
25	Germany	Side effects	1 in 10,000	0.00			
26	Germany	Side effects	1 in 100,000	0.27	0.05	0.17	0.36
27	Germany	Side effects	1 in 1,000,000	0.42	0.05	0.33	0.52
28	Germany	Effectiveness	55%	0.00			
29	Germany	Effectiveness	75%	0.54	0.05	0.44	0.63
30	Germany	Effectiveness	95%	1.01	0.05	0.91	1.12
31	Germany	Country	China	0.00			
32	Germany	Country	Russia	0.23	0.06	0.11	0.35
33	Germany	Country	Great Britain	0.62	0.06	0.49	0.74
34	Germany	Country	USA	0.65	0.07	0.52	0.78
35	Germany	Country	Germany	1.08	0.07	0.94	1.22
36	Germany	Type	Live Virus Vaccine	0.00			
37	Germany	Type	Viral Vector Vaccine	0.01	0.05	-0.09	0.11
38	Germany	Type	Subunit Vaccine	-0.04	0.05	-0.14	0.06
39	Germany	Type	mRNA Vaccine	0.15	0.06	0.04	0.25
40	Germany	People	1 million	0.00			
41	Germany	People	10 million	0.06	0.04	-0.03	0.15
42	Germany	People	100 million	0.21	0.05	0.13	0.30
43	Germany	duration	In 9 months	0.00			
44	Germany	duration	In 6 months	0.02	0.04	-0.07	0.11
45	Germany	duration	In 3 months	0.08	0.05	-0.01	0.17
46	Germany	cost	100 x population EUR/SEK/HUF	0.00			
47	Germany	cost	50 x population EUR/SEK/HUF	-0.03	0.04	-0.11	0.06
48	Germany	cost	10 x population EUR/SEK/HUF	0.03	0.04	-0.06	0.12
49	Sweden	Side effects	1 in 10,000	0.00			
50	Sweden	Side effects	1 in 100,000	0.23	0.04	0.14	0.32
51	Sweden	Side effects	1 in 1,000,000	0.41	0.05	0.32	0.50
52	Sweden	Effectiveness	55%	0.00			
53	Sweden	Effectiveness	75%	0.38	0.04	0.29	0.47
54	Sweden	Effectiveness	95%	0.84	0.05	0.74	0.94
55	Sweden	Country	China	0.00			
56	Sweden	Country	Russia	0.03	0.06	-0.08	0.14
57	Sweden	Country	Great Britain	0.71	0.06	0.58	0.84
58	Sweden	Country	USA	0.70	0.06	0.58	0.82
59	Sweden	Country	Germany	0.73	0.06	0.61	0.85
60	Sweden	Type	Live Virus Vaccine	0.00			
61	Sweden	Type	Viral Vector Vaccine	0.10	0.05	-0.00	0.21
62	Sweden	Type	Subunit Vaccine	0.08	0.05	-0.02	0.19
63	Sweden	Type	mRNA Vaccine	0.12	0.05	0.01	0.22
64	Sweden	People	1 million	0.00			
65	Sweden	People	10 million	0.06	0.04	-0.02	0.15
66	Sweden	People	100 million	0.17	0.04	0.09	0.26
67	Sweden	duration	In 9 months	0.00			
68	Sweden	duration	In 6 months	0.10	0.04	0.02	0.19
69	Sweden	duration	In 3 months	0.08	0.04	-0.00	0.17
70	Sweden	cost	100 x population EUR/SEK/HUF	0.00			
71	Sweden	cost	50 x population EUR/SEK/HUF	0.07	0.04	-0.01	0.16
72	Sweden	cost	10 x population EUR/SEK/HUF	0.08	0.04	-0.00	0.17

Table SI14: Cross-country MMs for vaccine choice

	Country	Attribute	Level	Estimate	SE	Lower CI	Upper CI
1	France	Side effects	1 in 10 000	0.43	0.00	0.42	0.44
2	France	Side effects	1 in 100 000	0.51	0.00	0.50	0.52
3	France	Side effects	1 in 1 000 000	0.56	0.00	0.55	0.57
4	France	Effectiveness	55%	0.35	0.00	0.34	0.36
5	France	Effectiveness	75%	0.52	0.00	0.51	0.52
6	France	Effectiveness	95%	0.64	0.00	0.63	0.65
7	France	Country	China	0.39	0.01	0.38	0.41
8	France	Country	Russia	0.45	0.01	0.43	0.46
9	France	Country	Great Britain	0.53	0.01	0.51	0.54
10	France	Country	USA	0.56	0.01	0.55	0.58
11	France	Country	Germany	0.57	0.01	0.56	0.58
12	France	Type	Live Virus Vaccine	0.48	0.01	0.47	0.49
13	France	Type	Viral Vector Vaccine	0.49	0.01	0.48	0.50
14	France	Type	Subunit Vaccine	0.50	0.01	0.49	0.51
15	France	Type	mRNA Vaccine	0.53	0.01	0.52	0.54
16	France	People	1 million	0.47	0.00	0.47	0.48
17	France	People	10 million	0.50	0.00	0.49	0.51
18	France	People	100 million	0.52	0.00	0.52	0.53
19	France	Duration	In 9 months	0.48	0.00	0.47	0.49
20	France	Duration	In 6 months	0.50	0.00	0.50	0.51
21	France	Duration	In 3 months	0.52	0.00	0.51	0.52
22	France	Cost	100 x population EUR/SEK/HUF	0.49	0.00	0.48	0.50
23	France	Cost	50 x population EUR/SEK/HUF	0.50	0.00	0.49	0.51
24	France	Cost	10 x population EUR/SEK/HUF	0.51	0.00	0.50	0.52
25	Germany	Side effects	1 in 10 000	0.44	0.00	0.43	0.45
26	Germany	Side effects	1 in 100 000	0.50	0.00	0.49	0.51
27	Germany	Side effects	1 in 1 000 000	0.56	0.00	0.55	0.57
28	Germany	Effectiveness	55%	0.36	0.00	0.35	0.37
29	Germany	Effectiveness	75%	0.51	0.00	0.50	0.51
30	Germany	Effectiveness	95%	0.63	0.00	0.62	0.64
31	Germany	Country	China	0.40	0.01	0.39	0.41
32	Germany	Country	Russia	0.45	0.01	0.44	0.46
33	Germany	Country	Great Britain	0.52	0.01	0.50	0.53
34	Germany	Country	USA	0.51	0.01	0.50	0.52
35	Germany	Country	Germany	0.62	0.01	0.61	0.63
36	Germany	Type	Live Virus Vaccine	0.49	0.01	0.48	0.50
37	Germany	Type	Viral Vector Vaccine	0.49	0.01	0.48	0.50
38	Germany	Type	Subunit Vaccine	0.49	0.01	0.48	0.50
39	Germany	Type	mRNA Vaccine	0.53	0.01	0.52	0.54
40	Germany	People	1 million	0.47	0.00	0.46	0.48
41	Germany	People	10 million	0.50	0.00	0.49	0.51
42	Germany	People	100 million	0.53	0.00	0.52	0.54
43	Germany	Duration	In 9 months	0.48	0.00	0.47	0.49
44	Germany	Duration	In 6 months	0.50	0.00	0.49	0.51
45	Germany	Duration	In 3 months	0.52	0.00	0.51	0.53
46	Germany	Cost	100 x population EUR/SEK/HUF	0.48	0.00	0.48	0.49
47	Germany	Cost	50 x population EUR/SEK/HUF	0.50	0.00	0.49	0.50
48	Germany	Cost	10 x population EUR/SEK/HUF	0.52	0.00	0.51	0.53
49	Sweden	Side effects	1 in 10 000	0.42	0.00	0.41	0.43
50	Sweden	Side effects	1 in 100 000	0.50	0.00	0.50	0.51
51	Sweden	Side effects	1 in 1 000 000	0.58	0.00	0.57	0.59
52	Sweden	Effectiveness	55%	0.36	0.00	0.35	0.37
53	Sweden	Effectiveness	75%	0.51	0.00	0.50	0.52
54	Sweden	Effectiveness	95%	0.63	0.00	0.62	0.64
55	Sweden	Country	China	0.41	0.01	0.40	0.42
56	Sweden	Country	Russia	0.42	0.01	0.41	0.44
57	Sweden	Country	Great Britain	0.55	0.01	0.54	0.56
58	Sweden	Country	USA	0.55	0.01	0.54	0.56
59	Sweden	Country	Germany	0.57	0.01	0.56	0.58
60	Sweden	Type	Live Virus Vaccine	0.50	0.01	0.49	0.51
61	Sweden	Type	Viral Vector Vaccine	0.49	0.01	0.48	0.50
62	Sweden	Type	Subunit Vaccine	0.50	0.01	0.49	0.51
63	Sweden	Type	mRNA Vaccine	0.51	0.01	0.49	0.52
64	Sweden	People	1 million	0.47	0.00	0.46	0.48
65	Sweden	People	10 million	0.50	0.00	0.50	0.51
66	Sweden	People	100 million	0.53	0.00	0.52	0.53
67	Sweden	Duration	In 9 months	0.47	0.00	0.46	0.48
68	Sweden	Duration	In 6 months	0.50	0.00	0.49	0.51
69	Sweden	Duration	In 3 months	0.53	0.00	0.52	0.54
70	Sweden	Cost	100 x population EUR/SEK/HUF	0.49	0.00	0.48	0.50
71	Sweden	Cost	50 x population EUR/SEK/HUF	0.50	0.00	0.49	0.51
72	Sweden	Cost	10 x population EUR/SEK/HUF	0.51	0.00	0.50	0.52

Table SI15: Cross-country AMCEs for vaccine choice

	Country	Attribute	Level	Estimate	SE	Lower CI	Upper CI
1	France	Side effects	1 in 10,000	0.00			
2	France	Side effects	1 in 100,000	0.08	0.01	0.06	0.09
3	France	Side effects	1 in 1,000,000	0.12	0.01	0.11	0.14
4	France	Effectiveness	55%	0.00			
5	France	Effectiveness	75%	0.17	0.01	0.15	0.18
6	France	Effectiveness	95%	0.29	0.01	0.27	0.30
7	France	Country	China	0.00			
8	France	Country	Russia	0.05	0.01	0.03	0.07
9	France	Country	Great Britain	0.14	0.01	0.12	0.16
10	France	Country	USA	0.17	0.01	0.15	0.19
11	France	Country	Germany	0.17	0.01	0.15	0.19
12	France	Type	Live Virus Vaccine	0.00			
13	France	Type	Viral Vector Vaccine	0.01	0.01	-0.00	0.03
14	France	Type	Subunit Vaccine	0.02	0.01	0.01	0.04
15	France	Type	mRNA Vaccine	0.05	0.01	0.03	0.07
16	France	People	1 million	0.00			
17	France	People	10 million	0.03	0.01	0.01	0.04
18	France	People	100 million	0.05	0.01	0.04	0.07
19	France	Duration	In 9 months	0.00			
20	France	Duration	In 6 months	0.02	0.01	0.01	0.04
21	France	Duration	In 3 months	0.04	0.01	0.02	0.05
22	France	Cost	100 x population EUR/SEK/HUF	0.00			
23	France	Cost	50 x population EUR/SEK/HUF	0.01	0.01	-0.00	0.02
24	France	Cost	10 x population EUR/SEK/HUF	0.02	0.01	0.01	0.03
25	germany	Side effects	1 in 10,000	0.00			
26	germany	Side effects	1 in 100,000	0.07	0.01	0.05	0.08
27	germany	Side effects	1 in 1,000,000	0.13	0.01	0.11	0.14
28	germany	Effectiveness	55%	0.00			
29	germany	Effectiveness	75%	0.14	0.01	0.13	0.16
30	germany	Effectiveness	95%	0.27	0.01	0.25	0.29
31	germany	Country	China	0.00			
32	germany	Country	Russia	0.05	0.01	0.03	0.07
33	germany	Country	Great Britain	0.12	0.01	0.10	0.14
34	germany	Country	USA	0.11	0.01	0.09	0.13
35	germany	Country	Germany	0.22	0.01	0.20	0.24
36	germany	Type	Live Virus Vaccine	0.00			
37	germany	Type	Viral Vector Vaccine	0.01	0.01	-0.01	0.03
38	germany	Type	Subunit Vaccine	0.00	0.01	-0.01	0.02
39	germany	Type	mRNA Vaccine	0.05	0.01	0.03	0.06
40	germany	People	1 million	0.00			
41	germany	People	10 million	0.03	0.01	0.02	0.04
42	germany	People	100 million	0.06	0.01	0.05	0.08
43	germany	Duration	In 9 months	0.00			
44	germany	Duration	In 6 months	0.02	0.01	0.01	0.04
45	germany	Duration	In 3 months	0.04	0.01	0.03	0.06
46	germany	Cost	100 x population EUR/SEK/HUF	0.00			
47	germany	Cost	50 x population EUR/SEK/HUF	0.01	0.01	-0.00	0.03
48	germany	Cost	10 x population EUR/SEK/HUF	0.04	0.01	0.02	0.05
49	Sweden	Side effects	1 in 10,000	0.00			
50	Sweden	Side effects	1 in 100,000	0.08	0.01	0.07	0.10
51	Sweden	Side effects	1 in 1,000,000	0.16	0.01	0.14	0.17
52	Sweden	Effectiveness	55%	0.00			
53	Sweden	Effectiveness	75%	0.15	0.01	0.13	0.16
54	Sweden	Effectiveness	95%	0.27	0.01	0.25	0.28
55	Sweden	Country	China	0.00			
56	Sweden	Country	Russia	0.01	0.01	-0.01	0.03
57	Sweden	Country	Great Britain	0.14	0.01	0.12	0.16
58	Sweden	Country	USA	0.13	0.01	0.12	0.15
59	Sweden	Country	Germany	0.16	0.01	0.14	0.18
60	Sweden	Type	Live Virus Vaccine	0.00			
61	Sweden	Type	Viral Vector Vaccine	-0.00	0.01	-0.02	0.01
62	Sweden	Type	Subunit Vaccine	-0.00	0.01	-0.02	0.01
63	Sweden	Type	mRNA Vaccine	0.00	0.01	-0.01	0.02
64	Sweden	People	1 million	0.00			
65	Sweden	People	10 million	0.03	0.01	0.02	0.05
66	Sweden	People	100 million	0.06	0.01	0.04	0.07
67	Sweden	Duration	In 9 months	0.00			
68	Sweden	Duration	In 6 months	0.02	0.01	0.01	0.04
69	Sweden	Duration	In 3 months	0.05	0.01	0.04	0.07
70	Sweden	Cost	100 x population EUR/SEK/HUF	0.00			
71	Sweden	Cost	50 x population EUR/SEK/HUF	0.02	0.01	0.00	0.03
72	Sweden	Cost	10 x population EUR/SEK/HUF	0.02	0.01	0.01	0.03

Table SI16: Vaccine hesitancy subgroup analysis: MMs for likelihood of uptake

	Subgroup	Attribute	Level	Estimate	SE	Lower CI	Upper CI
1	Vaccine supporters	Side effects	1 in 10,000	5.64	0.05	5.53	5.74
2	Vaccine supporters	Side effects	1 in 100,000	5.95	0.05	5.85	6.05
3	Vaccine supporters	Side effects	1 in 1,000,000	6.19	0.05	6.09	6.29
4	Vaccine supporters	Effectiveness	55%	5.30	0.05	5.19	5.40
5	Vaccine supporters	Effectiveness	75%	5.97	0.05	5.86	6.07
6	Vaccine supporters	Effectiveness	95%	6.52	0.05	6.41	6.62
7	Vaccine supporters	Country	China	5.22	0.06	5.09	5.34
8	Vaccine supporters	Country	Russia	5.42	0.06	5.30	5.54
9	Vaccine supporters	Country	Great Britain	6.22	0.06	6.11	6.33
10	Vaccine supporters	Country	USA	6.25	0.06	6.14	6.36
11	Vaccine supporters	Country	Germany	6.56	0.05	6.45	6.66
12	Vaccine supporters	Type	Live Virus Vaccine	5.81	0.06	5.70	5.92
13	Vaccine supporters	Type	Viral Vector Vaccine	5.92	0.05	5.82	6.03
14	Vaccine supporters	Type	Subunit Vaccine	5.86	0.05	5.75	5.96
15	Vaccine supporters	Type	mRNA Vaccine	6.11	0.05	6.01	6.22
16	Vaccine supporters	People	1 million	5.84	0.05	5.74	5.94
17	Vaccine supporters	People	10 million	5.90	0.05	5.80	6.00
18	Vaccine supporters	People	100 million	6.04	0.05	5.94	6.14
19	Vaccine supporters	Duration	In 9 months	5.83	0.05	5.73	5.94
20	Vaccine supporters	Duration	In 6 months	5.96	0.05	5.86	6.06
21	Vaccine supporters	Duration	In 3 months	5.98	0.05	5.88	6.08
22	Vaccine supporters	Cost	100 x population EUR/SEK/HUF	5.88	0.05	5.77	5.98
23	Vaccine supporters	Cost	50 x population EUR/SEK/HUF	5.88	0.05	5.78	5.98
24	Vaccine supporters	Cost	10 x population EUR/SEK/HUF	6.02	0.05	5.92	6.12
25	Vaccine acceptors	Side effects	1 in 10,000	4.66	0.05	4.55	4.76
26	Vaccine acceptors	Side effects	1 in 100,000	4.86	0.05	4.75	4.96
27	Vaccine acceptors	Side effects	1 in 1,000,000	5.01	0.05	4.90	5.11
28	Vaccine acceptors	Effectiveness	55%	4.43	0.05	4.33	4.54
29	Vaccine acceptors	Effectiveness	75%	4.82	0.05	4.71	4.93
30	Vaccine acceptors	Effectiveness	95%	5.27	0.06	5.16	5.38
31	Vaccine acceptors	Country	China	4.37	0.06	4.25	4.49
32	Vaccine acceptors	Country	Russia	4.51	0.06	4.39	4.63
33	Vaccine acceptors	Country	Great Britain	5.06	0.06	4.94	5.17
34	Vaccine acceptors	Country	USA	5.05	0.06	4.94	5.17
35	Vaccine acceptors	Country	Germany	5.20	0.06	5.09	5.32
36	Vaccine acceptors	Type	Live Virus Vaccine	4.78	0.06	4.67	4.89
37	Vaccine acceptors	Type	Viral Vector Vaccine	4.82	0.06	4.71	4.93
38	Vaccine acceptors	Type	Subunit Vaccine	4.85	0.06	4.74	4.95
39	Vaccine acceptors	Type	mRNA Vaccine	4.91	0.06	4.80	5.02
40	Vaccine acceptors	People	1 million	4.75	0.05	4.64	4.85
41	Vaccine acceptors	People	10 million	4.86	0.05	4.75	4.96
42	Vaccine acceptors	People	100 million	4.91	0.05	4.81	5.02
43	Vaccine acceptors	Duration	In 9 months	4.80	0.05	4.69	4.90
44	Vaccine acceptors	Duration	In 6 months	4.84	0.05	4.73	4.94
45	Vaccine acceptors	Duration	In 3 months	4.88	0.05	4.78	4.99
46	Vaccine acceptors	Cost	100 x population EUR/SEK/HUF	4.82	0.05	4.72	4.93
47	Vaccine acceptors	Cost	50 x population EUR/SEK/HUF	4.86	0.05	4.75	4.96
48	Vaccine acceptors	Cost	10 x population EUR/SEK/HUF	4.84	0.05	4.73	4.94
49	Vaccine hesitants	Side effects	1 in 10,000	4.31	0.08	4.14	4.47
50	Vaccine hesitants	Side effects	1 in 100,000	4.45	0.08	4.28	4.61
51	Vaccine hesitants	Side effects	1 in 1,000,000	4.39	0.08	4.23	4.56
52	Vaccine hesitants	Effectiveness	55%	4.15	0.08	3.98	4.31
53	Vaccine hesitants	Effectiveness	75%	4.38	0.08	4.22	4.54
54	Vaccine hesitants	Effectiveness	95%	4.62	0.08	4.46	4.79
55	Vaccine hesitants	Country	China	4.20	0.09	4.02	4.39
56	Vaccine hesitants	Country	Russia	4.35	0.09	4.17	4.52
57	Vaccine hesitants	Country	Great Britain	4.41	0.09	4.24	4.59
58	Vaccine hesitants	Country	USA	4.47	0.09	4.30	4.64
59	Vaccine hesitants	Country	Germany	4.48	0.09	4.30	4.66
60	Vaccine hesitants	Type	Live Virus Vaccine	4.39	0.09	4.22	4.57
61	Vaccine hesitants	Type	Viral Vector Vaccine	4.35	0.09	4.18	4.52
62	Vaccine hesitants	Type	Subunit Vaccine	4.37	0.09	4.20	4.54
63	Vaccine hesitants	Type	mRNA Vaccine	4.42	0.09	4.25	4.59
64	Vaccine hesitants	People	1 million	4.33	0.08	4.16	4.49
65	Vaccine hesitants	People	10 million	4.34	0.08	4.18	4.50
66	Vaccine hesitants	People	100 million	4.48	0.08	4.32	4.64
67	Vaccine hesitants	Duration	In 9 months	4.36	0.08	4.20	4.52
68	Vaccine hesitants	Duration	In 6 months	4.44	0.08	4.28	4.60
69	Vaccine hesitants	Duration	In 3 months	4.35	0.08	4.19	4.51
70	Vaccine hesitants	Cost	100 x population EUR/SEK/HUF	4.39	0.08	4.22	4.55
71	Vaccine hesitants	Cost	50 x population EUR/SEK/HUF	4.34	0.08	4.18	4.51
72	Vaccine hesitants	Cost	10 x population EUR/SEK/HUF	4.42	0.08	4.26	4.58

Table SI17: Vaccine hesitancy subgroup analysis: AMCEs for likelihood of uptake

	Subgroup	Attribute	Level	Estimate	SE	Lower CI	Upper CI
1	Vaccine supporters	Side effects	1 in 10,000	0.00			
2	Vaccine supporters	Side effects	1 in 100,000	0.30	0.04	0.22	0.38
3	Vaccine supporters	Side effects	1 in 1,000,000	0.55	0.04	0.46	0.64
4	Vaccine supporters	Effective	55%	0.00			
5	Vaccine supporters	Effective	75%	0.67	0.05	0.59	0.76
6	Vaccine supporters	Effective	95%	1.21	0.05	1.11	1.31
7	Vaccine supporters	Country	China	0.00			
8	Vaccine supporters	Country	Russia	0.20	0.06	0.09	0.31
9	Vaccine supporters	Country	Great Britain	1.01	0.06	0.89	1.14
10	Vaccine supporters	Country	USA	1.03	0.06	0.90	1.15
11	Vaccine supporters	Country	Germany	1.35	0.06	1.22	1.47
12	Vaccine supporters	Type	Live Virus Vaccine	0.00			
13	Vaccine supporters	Type	Viral Vector Vaccine	0.11	0.05	0.02	0.21
14	Vaccine supporters	Type	Subunit Vaccine	0.05	0.05	-0.05	0.14
15	Vaccine supporters	Type	mRNA Vaccine	0.28	0.05	0.18	0.38
16	Vaccine supporters	People	1 million	0.00			
17	Vaccine supporters	People	10 million	0.08	0.04	-0.00	0.16
18	Vaccine supporters	People	100 million	0.22	0.04	0.13	0.30
19	Vaccine supporters	Duration	In 9 months	0.00			
20	Vaccine supporters	Duration	In 6 months	0.11	0.04	0.02	0.19
21	Vaccine supporters	Duration	In 3 months	0.14	0.04	0.06	0.23
22	Vaccine supporters	Cost	100 x population EUR/SEK/HUF	0.00			
23	Vaccine supporters	Cost	50 x population EUR/SEK/HUF	0.02	0.04	-0.06	0.09
24	Vaccine supporters	Cost	10 x population EUR/SEK/HUF	0.15	0.04	0.06	0.23
25	Vaccine acceptors	Side effects	1 in 10,000	0.00			
26	Vaccine acceptors	Side effects	1 in 100,000	0.19	0.04	0.11	0.26
27	Vaccine acceptors	Side effects	1 in 1,000,000	0.34	0.04	0.25	0.42
28	Vaccine acceptors	Effective	55%	0.00			
29	Vaccine acceptors	Effective	75%	0.40	0.04	0.32	0.48
30	Vaccine acceptors	Effective	95%	0.84	0.05	0.75	0.93
31	Vaccine acceptors	Country	China	0.00			
32	Vaccine acceptors	Country	Russia	0.15	0.05	0.05	0.26
33	Vaccine acceptors	Country	Great Britain	0.72	0.06	0.61	0.82
34	Vaccine acceptors	Country	USA	0.69	0.06	0.58	0.80
35	Vaccine acceptors	Country	Germany	0.82	0.06	0.71	0.94
36	Vaccine acceptors	Type	Live Virus Vaccine	0.00			
37	Vaccine acceptors	Type	Viral Vector Vaccine	0.06	0.05	-0.03	0.15
38	Vaccine acceptors	Type	Subunit Vaccine	0.08	0.04	-0.01	0.16
39	Vaccine acceptors	Type	mRNA Vaccine	0.16	0.05	0.06	0.25
40	Vaccine acceptors	People	1 million	0.00			
41	Vaccine acceptors	People	10 million	0.12	0.04	0.04	0.19
42	Vaccine acceptors	People	100 million	0.18	0.04	0.10	0.26
43	Vaccine acceptors	Duration	In 9 months	0.00			
44	Vaccine acceptors	Duration	In 6 months	0.06	0.04	-0.02	0.13
45	Vaccine acceptors	Duration	In 3 months	0.10	0.04	0.03	0.18
46	Vaccine acceptors	Cost	100 x population EUR/SEK/HUF	0.00			
47	Vaccine acceptors	Cost	50 x population EUR/SEK/HUF	0.02	0.04	-0.06	0.10
48	Vaccine acceptors	Cost	10 x population EUR/SEK/HUF	0.01	0.04	-0.07	0.08
49	Vaccine hesitants	Side effects	1 in 10,000	0.00			
50	Vaccine hesitants	Side effects	1 in 100,000	0.14	0.06	0.03	0.25
51	Vaccine hesitants	Side effects	1 in 1,000,000	0.08	0.05	-0.03	0.18
52	Vaccine hesitants	Effective	55%	0.00			
53	Vaccine hesitants	Effective	75%	0.24	0.05	0.14	0.34
54	Vaccine hesitants	Effective	95%	0.48	0.06	0.38	0.59
55	Vaccine hesitants	Country	China	0.00			
56	Vaccine hesitants	Country	Russia	0.15	0.07	0.01	0.28
57	Vaccine hesitants	Country	Great Britain	0.20	0.07	0.06	0.34
58	Vaccine hesitants	Country	USA	0.26	0.07	0.13	0.40
59	Vaccine hesitants	Country	Germany	0.26	0.07	0.12	0.41
60	Vaccine hesitants	Type	Live Virus Vaccine	0.00			
61	Vaccine hesitants	Type	Viral Vector Vaccine	-0.04	0.06	-0.16	0.09
62	Vaccine hesitants	Type	Subunit Vaccine	-0.01	0.06	-0.13	0.11
63	Vaccine hesitants	Type	mRNA Vaccine	0.03	0.06	-0.10	0.15
64	Vaccine hesitants	People	1 million	0.00			
65	Vaccine hesitants	People	10 million	-0.01	0.05	-0.11	0.10
66	Vaccine hesitants	People	100 million	0.13	0.05	0.03	0.23
67	Vaccine hesitants	Duration	In 9 months	0.00			
68	Vaccine hesitants	Duration	In 6 months	0.07	0.05	-0.03	0.17
69	Vaccine hesitants	Duration	In 3 months	-0.01	0.05	-0.11	0.09
70	Vaccine hesitants	Cost	100 x population EUR/SEK/HUF	0.00			
71	Vaccine hesitants	Cost	50 x population EUR/SEK/HUF	-0.02	0.05	-0.12	0.08
72	Vaccine hesitants	Cost	10 x population EUR/SEK/HUF	0.05	0.05	-0.05	0.15

Figure SI6: Subgroup analysis: Differences across different vaccine hesitant groups

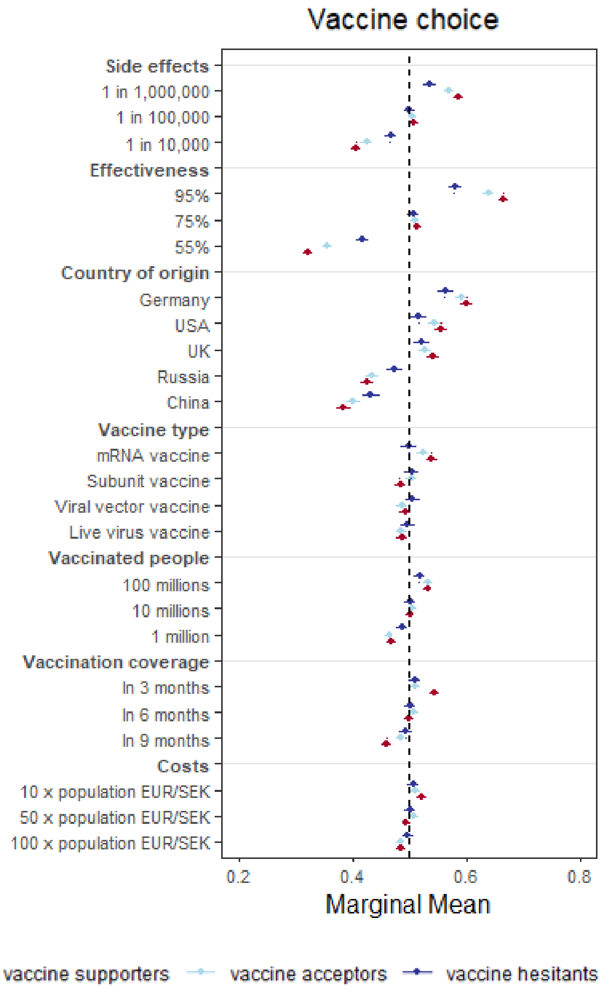

Note. MMs of vaccine attributes on vaccine choice for the three groups of people with similar profiles of vaccine attitudes. For the side effects, effectiveness, and country of origin attribute, we find that risk-averse respondents tend to more strongly discriminate between attribute levels (e.g., 55% vs. 95% effectiveness) than vaccine acceptors, and the vaccine hesitant.

Table SI18: Vaccine hesitancy subgroup analysis: MMs for vaccine choice

	Subgroup	Attribute	Level	Estimate	SE	Lower CI	Upper CI
1	Vaccine supporters	Side effects	1 in 10,000	5.63	0.05	5.53	5.73
2	Vaccine supporters	Side effects	1 in 100,000	5.94	0.05	5.84	6.03
3	Vaccine supporters	Side effects	1 in 1,000,000	6.19	0.05	6.09	6.29
4	Vaccine supporters	Effectiveness	55%	5.29	0.05	5.19	5.40
5	Vaccine supporters	Effectiveness	75%	5.96	0.05	5.86	6.06
6	Vaccine supporters	Effectiveness	95%	6.50	0.05	6.40	6.60
7	Vaccine supporters	Country	China	5.21	0.06	5.08	5.33
8	Vaccine supporters	Country	Russia	5.41	0.06	5.29	5.54
9	Vaccine supporters	Country	Great Britain	6.21	0.06	6.10	6.32
10	Vaccine supporters	Country	USA	6.24	0.06	6.13	6.35
11	Vaccine supporters	Country	Germany	6.55	0.05	6.45	6.66
12	Vaccine supporters	Type	Live Virus Vaccine	5.81	0.06	5.70	5.92
13	Vaccine supporters	Type	Viral Vector Vaccine	5.91	0.05	5.81	6.02
14	Vaccine supporters	Type	Subunit Vaccine	5.84	0.05	5.74	5.95
15	Vaccine supporters	Type	mRNA Vaccine	6.11	0.05	6.01	6.21
16	Vaccine supporters	People	1 million	5.83	0.05	5.73	5.93
17	Vaccine supporters	People	10 million	5.89	0.05	5.79	5.99
18	Vaccine supporters	People	100 million	6.03	0.05	5.94	6.13
19	Vaccine supporters	Duration	In 9 months	5.83	0.05	5.73	5.93
20	Vaccine supporters	Duration	In 6 months	5.95	0.05	5.85	6.05
21	Vaccine supporters	Duration	In 3 months	5.98	0.05	5.88	6.08
22	Vaccine supporters	Cost	100 x population EUR/SEK/HUF	5.87	0.05	5.77	5.97
23	Vaccine supporters	Cost	50 x population EUR/SEK/HUF	5.88	0.05	5.78	5.98
24	Vaccine supporters	Cost	10 x population EUR/SEK/HUF	6.01	0.05	5.91	6.11
25	Vaccine acceptors	Side effects	1 in 10,000	4.65	0.05	4.55	4.76
26	Vaccine acceptors	Side effects	1 in 100,000	4.85	0.05	4.75	4.95
27	Vaccine acceptors	Side effects	1 in 1,000,000	5.00	0.05	4.90	5.11
28	Vaccine acceptors	Effectiveness	55%	4.42	0.05	4.32	4.53
29	Vaccine acceptors	Effectiveness	75%	4.83	0.05	4.72	4.93
30	Vaccine acceptors	Effectiveness	95%	5.27	0.06	5.16	5.37
31	Vaccine acceptors	Country	China	4.36	0.06	4.23	4.48
32	Vaccine acceptors	Country	Russia	4.51	0.06	4.40	4.63
33	Vaccine acceptors	Country	Great Britain	5.06	0.06	4.95	5.18
34	Vaccine acceptors	Country	USA	5.05	0.06	4.94	5.17
35	Vaccine acceptors	Country	Germany	5.19	0.06	5.07	5.30
36	Vaccine acceptors	Type	Live Virus Vaccine	4.77	0.06	4.66	4.88
37	Vaccine acceptors	Type	Viral Vector Vaccine	4.81	0.06	4.70	4.92
38	Vaccine acceptors	Type	Subunit Vaccine	4.85	0.05	4.74	4.95
39	Vaccine acceptors	Type	mRNA Vaccine	4.92	0.06	4.80	5.03
40	Vaccine acceptors	People	1 million	4.74	0.05	4.63	4.84
41	Vaccine acceptors	People	10 million	4.86	0.05	4.75	4.96
42	Vaccine acceptors	People	100 million	4.91	0.05	4.81	5.02
43	Vaccine acceptors	Duration	In 9 months	4.78	0.05	4.68	4.89
44	Vaccine acceptors	Duration	In 6 months	4.84	0.05	4.74	4.95
45	Vaccine acceptors	Duration	In 3 months	4.88	0.05	4.78	4.99
46	Vaccine acceptors	Cost	100 x population EUR/SEK/HUF	4.83	0.05	4.73	4.93
47	Vaccine acceptors	Cost	50 x population EUR/SEK/HUF	4.85	0.05	4.75	4.96
48	Vaccine acceptors	Cost	10 x population EUR/SEK/HUF	4.83	0.05	4.72	4.94
49	Vaccine hesitants	Side effects	1 in 10,000	4.30	0.08	4.13	4.46
50	Vaccine hesitants	Side effects	1 in 100,000	4.44	0.08	4.28	4.59
51	Vaccine hesitants	Side effects	1 in 1,000,000	4.38	0.08	4.22	4.54
52	Vaccine hesitants	Effectiveness	55%	4.13	0.08	3.97	4.29
53	Vaccine hesitants	Effectiveness	75%	4.37	0.08	4.21	4.52
54	Vaccine hesitants	Effectiveness	95%	4.61	0.08	4.45	4.77
55	Vaccine hesitants	Country	China	4.19	0.09	4.02	4.37
56	Vaccine hesitants	Country	Russia	4.34	0.09	4.17	4.51
57	Vaccine hesitants	Country	Great Britain	4.39	0.09	4.22	4.56
58	Vaccine hesitants	Country	USA	4.46	0.08	4.30	4.63
59	Vaccine hesitants	Country	Germany	4.46	0.09	4.28	4.63
60	Vaccine hesitants	Type	Live Virus Vaccine	4.37	0.09	4.20	4.54
61	Vaccine hesitants	Type	Viral Vector Vaccine	4.34	0.08	4.17	4.50
62	Vaccine hesitants	Type	Subunit Vaccine	4.37	0.08	4.21	4.53
63	Vaccine hesitants	Type	mRNA Vaccine	4.40	0.08	4.24	4.57
64	Vaccine hesitants	People	1 million	4.33	0.08	4.17	4.49
65	Vaccine hesitants	People	10 million	4.32	0.08	4.16	4.48
66	Vaccine hesitants	People	100 million	4.46	0.08	4.30	4.61
67	Vaccine hesitants	Duration	In 9 months	4.35	0.08	4.19	4.51
68	Vaccine hesitants	Duration	In 6 months	4.42	0.08	4.26	4.58
69	Vaccine hesitants	Duration	In 3 months	4.34	0.08	4.18	4.50
70	Vaccine hesitants	Cost	100 x population EUR/SEK/HUF	4.36	0.08	4.20	4.52
71	Vaccine hesitants	Cost	50 x population EUR/SEK/HUF	4.34	0.08	4.18	4.50
72	Vaccine hesitants	Cost	10 x population EUR/SEK/HUF	4.41	0.08	4.25	4.57

Table SI19: Vaccine hesitancy subgroup analysis: AMCEs for vaccine choice

	Subgroup	Attribute	Level	Estimate	SE	Lower CI	Upper CI
1	Vaccine supporters	Side effects	1 in 10 000	0.00			
2	Vaccine supporters	Side effects	1 in 100 000	0.30	0.04	0.22	0.38
3	Vaccine supporters	Side effects	1 in 1 000 000	0.55	0.04	0.46	0.64
4	Vaccine supporters	Effectiveness	55%	0.00			
5	Vaccine supporters	Effectiveness	75%	0.67	0.05	0.59	0.76
6	Vaccine supporters	Effectiveness	95%	1.21	0.05	1.11	1.31
7	Vaccine supporters	Country	China	0.00			
8	Vaccine supporters	Country	Russia	0.20	0.06	0.09	0.31
9	Vaccine supporters	Country	Great Britain	1.01	0.06	0.89	1.14
10	Vaccine supporters	Country	USA	1.03	0.06	0.90	1.15
11	Vaccine supporters	Country	Germany	1.35	0.06	1.22	1.47
12	Vaccine supporters	Type	Live Virus Vaccine	0.00			
13	Vaccine supporters	Type	Viral Vector Vaccine	0.11	0.05	0.02	0.21
14	Vaccine supporters	Type	Subunit Vaccine	0.05	0.05	-0.05	0.14
15	Vaccine supporters	Type	mRNA Vaccine	0.28	0.05	0.18	0.38
16	Vaccine supporters	People	1 million	0.00			
17	Vaccine supporters	People	10 million	0.08	0.04	-0.00	0.16
18	Vaccine supporters	People	100 million	0.22	0.04	0.13	0.30
19	Vaccine supporters	Duration	In 9 months	0.00			
20	Vaccine supporters	Duration	In 6 months	0.11	0.04	0.02	0.19
21	Vaccine supporters	Duration	In 3 months	0.14	0.04	0.06	0.23
22	Vaccine supporters	Cost	100 x population EUR/SEK/HUF	0.00			
23	Vaccine supporters	Cost	50 x population EUR/SEK/HUF	0.02	0.04	-0.06	0.09
24	Vaccine supporters	Cost	10 x population EUR/SEK/HUF	0.15	0.04	0.06	0.23
25	Vaccine acceptors	Side effects	1 in 10 000	0.00			
26	Vaccine acceptors	Side effects	1 in 100 000	0.19	0.04	0.11	0.26
27	Vaccine acceptors	Side effects	1 in 1 000 000	0.34	0.04	0.25	0.42
28	Vaccine acceptors	Effectiveness	55%	0.00			
29	Vaccine acceptors	Effectiveness	75%	0.40	0.04	0.32	0.48
30	Vaccine acceptors	Effectiveness	95%	0.84	0.05	0.75	0.93
31	Vaccine acceptors	Country	China	0.00			
32	Vaccine acceptors	Country	Russia	0.15	0.05	0.05	0.26
33	Vaccine acceptors	Country	Great Britain	0.72	0.06	0.61	0.82
34	Vaccine acceptors	Country	USA	0.69	0.06	0.58	0.80
35	Vaccine acceptors	Country	Germany	0.82	0.06	0.71	0.94
36	Vaccine acceptors	Type	Live Virus Vaccine	0.00			
37	Vaccine acceptors	Type	Viral Vector Vaccine	0.06	0.05	-0.03	0.15
38	Vaccine acceptors	Type	Subunit Vaccine	0.08	0.04	-0.01	0.16
39	Vaccine acceptors	Type	mRNA Vaccine	0.16	0.05	0.06	0.25
40	Vaccine acceptors	People	1 million	0.00			
41	Vaccine acceptors	People	10 million	0.12	0.04	0.04	0.19
42	Vaccine acceptors	People	100 million	0.18	0.04	0.10	0.26
43	Vaccine acceptors	Duration	In 9 months	0.00			
44	Vaccine acceptors	Duration	In 6 months	0.06	0.04	-0.02	0.13
45	Vaccine acceptors	Duration	In 3 months	0.10	0.04	0.03	0.18
46	Vaccine acceptors	Cost	100 x population EUR/SEK/HUF	0.00			
47	Vaccine acceptors	Cost	50 x population EUR/SEK/HUF	0.02	0.04	-0.06	0.10
48	Vaccine acceptors	Cost	10 x population EUR/SEK/HUF	0.01	0.04	-0.07	0.08
49	Vaccine hesitants	Side effects	1 in 10 000	0.00			
50	Vaccine hesitants	Side effects	1 in 100 000	0.14	0.06	0.03	0.25
51	Vaccine hesitants	Side effects	1 in 1 000 000	0.08	0.05	-0.03	0.18
52	Vaccine hesitants	Effectiveness	55%	0.00			
53	Vaccine hesitants	Effectiveness	75%	0.24	0.05	0.14	0.34
54	Vaccine hesitants	Effectiveness	95%	0.48	0.06	0.38	0.59
55	Vaccine hesitants	Country	China	0.00			
56	Vaccine hesitants	Country	Russia	0.15	0.07	0.01	0.28
57	Vaccine hesitants	Country	Great Britain	0.20	0.07	0.06	0.34
58	Vaccine hesitants	Country	USA	0.26	0.07	0.13	0.40
59	Vaccine hesitants	Country	Germany	0.26	0.07	0.12	0.41
60	Vaccine hesitants	Type	Live Virus Vaccine	0.00			
61	Vaccine hesitants	Type	Viral Vector Vaccine	-0.04	0.06	-0.16	0.09
62	Vaccine hesitants	Type	Subunit Vaccine	-0.01	0.06	-0.13	0.11
63	Vaccine hesitants	Type	mRNA Vaccine	0.03	0.06	-0.10	0.15
64	Vaccine hesitants	People	1 million	0.00			
65	Vaccine hesitants	People	10 million	-0.01	0.05	-0.11	0.10
66	Vaccine hesitants	People	100 million	0.13	0.05	0.03	0.23
67	Vaccine hesitants	Duration	In 9 months	0.00			
68	Vaccine hesitants	Duration	In 6 months	0.07	0.05	-0.03	0.17
69	Vaccine hesitants	Duration	In 3 months	-0.01	0.05	-0.11	0.09
70	Vaccine hesitants	Cost	100 x population EUR/SEK/HUF	0.00			
71	Vaccine hesitants	Cost	50 x population EUR/SEK/HUF	-0.02	0.05	-0.12	0.08
72	Vaccine hesitants	Cost	10 x population EUR/SEK/HUF	0.05	0.05	-0.05	0.15

Table SI20: Risk preference subgroup analysis: MMs for likelihood of uptake

	Subgroup	Attribute	Level	Estimate	SE	Lower CI	Upper CI
1	Risk averse	Side effects	1 in 10,000	4.47	0.05	4.37	4.56
2	Risk averse	Side effects	1 in 100,000	4.74	0.05	4.65	4.84
3	Risk averse	Side effects	1 in 1,000,000	4.89	0.05	4.79	4.98
4	Risk averse	Effectiveness	55%	4.23	0.05	4.13	4.32
5	Risk averse	Effectiveness	75%	4.72	0.05	4.62	4.81
6	Risk averse	Effectiveness	95%	5.15	0.05	5.05	5.25
7	Risk averse	Country	China	4.20	0.05	4.10	4.31
8	Risk averse	Country	Russia	4.37	0.05	4.27	4.48
9	Risk averse	Country	Great Britain	4.86	0.05	4.75	4.96
10	Risk averse	Country	USA	4.95	0.05	4.84	5.05
11	Risk averse	Country	Germany	5.13	0.05	5.02	5.23
12	Risk averse	Type	Live Virus Vaccine	4.62	0.05	4.51	4.72
13	Risk averse	Type	Viral Vector Vaccine	4.66	0.05	4.56	4.76
14	Risk averse	Type	Subunit Vaccine	4.68	0.05	4.59	4.78
15	Risk averse	Type	mRNA Vaccine	4.84	0.05	4.74	4.94
16	Risk averse	People	1 million	4.61	0.05	4.51	4.71
17	Risk averse	People	10 million	4.69	0.05	4.59	4.78
18	Risk averse	People	100 million	4.80	0.05	4.71	4.90
19	Risk averse	Duration	In 9 months	4.66	0.05	4.57	4.76
20	Risk averse	Duration	In 6 months	4.70	0.05	4.61	4.80
21	Risk averse	Duration	In 3 months	4.73	0.05	4.64	4.83
22	Risk averse	Cost	100 x population EUR/SEK/HUF	4.67	0.05	4.58	4.77
23	Risk averse	Cost	50 x population EUR/SEK/HUF	4.68	0.05	4.59	4.78
24	Risk averse	Cost	10 x population EUR/SEK/HUF	4.74	0.05	4.65	4.84
25	Risk seeking	Side effects	1 in 10,000	5.49	0.05	5.39	5.59
26	Risk seeking	Side effects	1 in 100,000	5.63	0.05	5.54	5.73
27	Risk seeking	Side effects	1 in 1,000,000	5.77	0.05	5.67	5.87
28	Risk seeking	Effectiveness	55%	5.18	0.05	5.08	5.28
29	Risk seeking	Effectiveness	75%	5.65	0.05	5.56	5.75
30	Risk seeking	Effectiveness	95%	6.07	0.05	5.97	6.17
31	Risk seeking	Country	China	5.15	0.06	5.03	5.27
32	Risk seeking	Country	Russia	5.33	0.06	5.22	5.44
33	Risk seeking	Country	Great Britain	5.86	0.05	5.75	5.97
34	Risk seeking	Country	USA	5.83	0.05	5.73	5.94
35	Risk seeking	Country	Germany	5.99	0.06	5.88	6.10
36	Risk seeking	Type	Live Virus Vaccine	5.58	0.05	5.47	5.68
37	Risk seeking	Type	Viral Vector Vaccine	5.63	0.05	5.53	5.74
38	Risk seeking	Type	Subunit Vaccine	5.59	0.05	5.49	5.70
39	Risk seeking	Type	mRNA Vaccine	5.72	0.05	5.62	5.83
40	Risk seeking	People	1 million	5.54	0.05	5.44	5.64
41	Risk seeking	People	10 million	5.63	0.05	5.53	5.73
42	Risk seeking	People	100 million	5.73	0.05	5.63	5.83
43	Risk seeking	Duration	In 9 months	5.55	0.05	5.45	5.65
44	Risk seeking	Duration	In 6 months	5.68	0.05	5.58	5.78
45	Risk seeking	Duration	In 3 months	5.67	0.05	5.57	5.77
46	Risk seeking	Cost	100 x population EUR/SEK/HUF	5.60	0.05	5.50	5.70
47	Risk seeking	Cost	50 x population EUR/SEK/HUF	5.62	0.05	5.52	5.72
48	Risk seeking	Cost	10 x population EUR/SEK/HUF	5.67	0.05	5.57	5.77

Table SI21: Risk preference subgroup analysis: AMCEs for likelihood of uptake

	Subgroup	Attribute	Level	Estimate	SE	Lower CI	Upper CI
1	Risk averse	Side effects	1 in 10,000	0.00			
2	Risk averse	Side effects	1 in 100,000	0.27	0.04	0.20	0.34
3	Risk averse	Side effects	1 in 1,000,000	0.41	0.04	0.34	0.49
4	Risk averse	Effective	55%	0.00			
5	Risk averse	Effective	75%	0.50	0.04	0.42	0.57
6	Risk averse	Effective	95%	0.92	0.04	0.84	1.00
7	Risk averse	Country	China	0.00			
8	Risk averse	Country	Russia	0.16	0.05	0.07	0.25
9	Risk averse	Country	Great Britain	0.66	0.05	0.57	0.76
10	Risk averse	Country	USA	0.74	0.05	0.64	0.84
11	Risk averse	Country	Germany	0.92	0.05	0.82	1.02
12	Risk averse	Type	Live Virus Vaccine	0.00			
13	Risk averse	Type	Viral Vector Vaccine	0.06	0.04	-0.02	0.14
14	Risk averse	Type	Subunit Vaccine	0.07	0.04	-0.02	0.15
15	Risk averse	Type	mRNA Vaccine	0.23	0.04	0.14	0.31
16	Risk averse	People	1 million	0.00			
17	Risk averse	People	10 million	0.08	0.04	0.01	0.15
18	Risk averse	People	100 million	0.20	0.04	0.13	0.27
19	Risk averse	Duration	In 9 months	0.00			
20	Risk averse	Duration	In 6 months	0.04	0.04	-0.03	0.10
21	Risk averse	Duration	In 3 months	0.07	0.04	-0.00	0.14
22	Risk averse	Cost	100 x population EUR/SEK/HUF	0.00			
23	Risk averse	Cost	50 x population EUR/SEK/HUF	0.01	0.03	-0.06	0.07
24	Risk averse	Cost	10 x population EUR/SEK/HUF	0.07	0.04	-0.00	0.13
25	Risk seeking	Side effects	1 in 10,000	0.00			
26	Risk seeking	Side effects	1 in 100,000	0.14	0.04	0.07	0.21
27	Risk seeking	Side effects	1 in 1,000,000	0.27	0.04	0.19	0.34
28	Risk seeking	Effective	55%	0.00			
29	Risk seeking	Effective	75%	0.47	0.04	0.39	0.54
30	Risk seeking	Effective	95%	0.88	0.04	0.80	0.97
31	Risk seeking	Country	China	0.00			
32	Risk seeking	Country	Russia	0.18	0.05	0.09	0.28
33	Risk seeking	Country	Great Britain	0.71	0.05	0.61	0.82
34	Risk seeking	Country	USA	0.68	0.05	0.57	0.78
35	Risk seeking	Country	Germany	0.84	0.06	0.73	0.95
36	Risk seeking	Type	Live Virus Vaccine	0.00			
37	Risk seeking	Type	Viral Vector Vaccine	0.06	0.04	-0.03	0.14
38	Risk seeking	Type	Subunit Vaccine	0.01	0.04	-0.07	0.09
39	Risk seeking	Type	mRNA Vaccine	0.14	0.04	0.05	0.23
40	Risk seeking	People	1 million	0.00			
41	Risk seeking	People	10 million	0.09	0.04	0.02	0.16
42	Risk seeking	People	100 million	0.19	0.04	0.12	0.27
43	Risk seeking	Duration	In 9 months	0.00			
44	Risk seeking	Duration	In 6 months	0.12	0.04	0.04	0.19
45	Risk seeking	Duration	In 3 months	0.11	0.04	0.04	0.19
46	Risk seeking	Cost	100 x population EUR/SEK/HUF	0.00			
47	Risk seeking	Cost	50 x population EUR/SEK/HUF	0.03	0.04	-0.04	0.10
48	Risk seeking	Cost	10 x population EUR/SEK/HUF	0.07	0.04	0.00	0.14

Figure SI7: Subgroup analysis: Differences across different risk preference groups

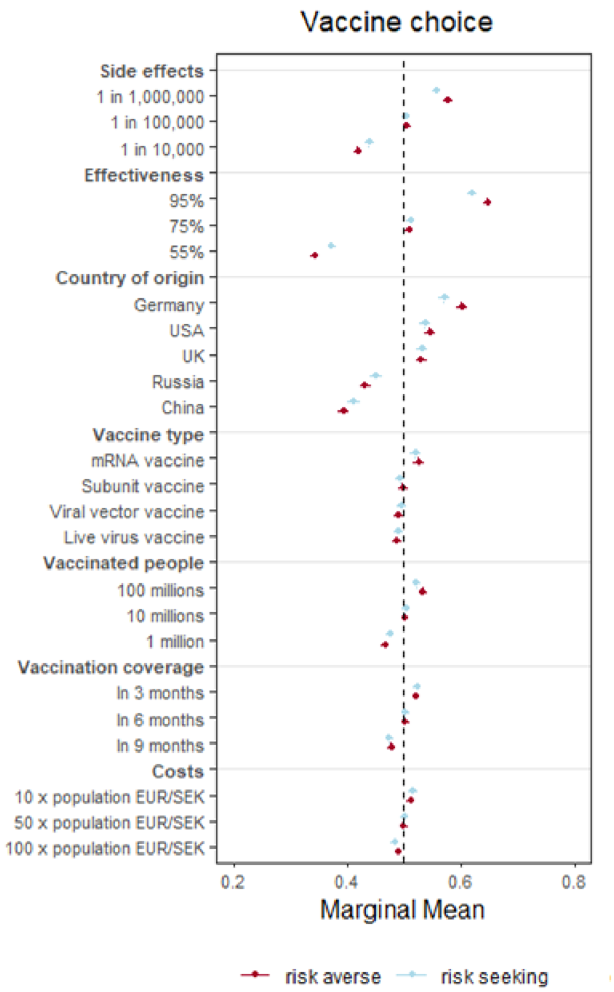

Note. MMs of vaccine attributes on vaccine choice. For the side effects, effectiveness, and country of origin attribute, we find risk-averse respondents tend to more strongly discriminate between attribute levels (e.g., 55% vs. 95% effectiveness) than vaccine acceptors, and the vaccine hesitant.

Table SI22: Risk preference subgroup analysis: MMs for vaccine choice

	Subgroup	Attribute	Level	Estimate	SE	Lower CI	Upper CI
1	Risk averse	Side effects	1 in 10,000	4.47	0.05	4.37	4.56
2	Risk averse	Side effects	1 in 100,000	4.74	0.05	4.65	4.84
3	Risk averse	Side effects	1 in 1,000,000	4.89	0.05	4.79	4.98
4	Risk averse	Effectiveness	55%	4.23	0.05	4.13	4.32
5	Risk averse	Effectiveness	75%	4.72	0.05	4.62	4.81
6	Risk averse	Effectiveness	95%	5.15	0.05	5.05	5.25
7	Risk averse	Country	China	4.20	0.05	4.10	4.31
8	Risk averse	Country	Russia	4.37	0.05	4.27	4.48
9	Risk averse	Country	Great Britain	4.86	0.05	4.75	4.96
10	Risk averse	Country	USA	4.95	0.05	4.84	5.05
11	Risk averse	Country	Germany	5.13	0.05	5.02	5.23
12	Risk averse	Type	Live Virus Vaccine	4.62	0.05	4.51	4.72
13	Risk averse	Type	Viral Vector Vaccine	4.66	0.05	4.56	4.76
14	Risk averse	Type	Subunit Vaccine	4.68	0.05	4.59	4.78
15	Risk averse	Type	mRNA Vaccine	4.84	0.05	4.74	4.94
16	Risk averse	People	1 million	4.61	0.05	4.51	4.71
17	Risk averse	People	10 million	4.69	0.05	4.59	4.78
18	Risk averse	People	100 million	4.80	0.05	4.71	4.90
19	Risk averse	Duration	In 9 months	4.66	0.05	4.57	4.76
20	Risk averse	Duration	In 6 months	4.70	0.05	4.61	4.80
21	Risk averse	Duration	In 3 months	4.73	0.05	4.64	4.83
22	Risk averse	Cost	100 x population EUR/SEK/HUF	4.67	0.05	4.58	4.77
23	Risk averse	Cost	50 x population EUR/SEK/HUF	4.68	0.05	4.59	4.78
24	Risk averse	Cost	10 x population EUR/SEK/HUF	4.74	0.05	4.65	4.84
25	Risk seeking	Side effects	1 in 10,000	5.49	0.05	5.39	5.59
26	Risk seeking	Side effects	1 in 100,000	5.63	0.05	5.54	5.73
27	Risk seeking	Side effects	1 in 1,000,000	5.77	0.05	5.67	5.87
28	Risk seeking	Effectiveness	55%	5.18	0.05	5.08	5.28
29	Risk seeking	Effectiveness	75%	5.65	0.05	5.56	5.75
30	Risk seeking	Effectiveness	95%	6.07	0.05	5.97	6.17
31	Risk seeking	Country	China	5.15	0.06	5.03	5.27
32	Risk seeking	Country	Russia	5.33	0.06	5.22	5.44
33	Risk seeking	Country	Great Britain	5.86	0.05	5.75	5.97
34	Risk seeking	Country	USA	5.83	0.05	5.73	5.94
35	Risk seeking	Country	Germany	5.99	0.06	5.88	6.10
36	Risk seeking	Type	Live Virus Vaccine	5.58	0.05	5.47	5.68
37	Risk seeking	Type	Viral Vector Vaccine	5.63	0.05	5.53	5.74
38	Risk seeking	Type	Subunit Vaccine	5.59	0.05	5.49	5.70
39	Risk seeking	Type	mRNA Vaccine	5.72	0.05	5.62	5.83
40	Risk seeking	People	1 million	5.54	0.05	5.44	5.64
41	Risk seeking	People	10 million	5.63	0.05	5.53	5.73
42	Risk seeking	People	100 million	5.73	0.05	5.63	5.83
43	Risk seeking	Duration	In 9 months	5.55	0.05	5.45	5.65
44	Risk seeking	Duration	In 6 months	5.68	0.05	5.58	5.78
45	Risk seeking	Duration	In 3 months	5.67	0.05	5.57	5.77
46	Risk seeking	Cost	100 x population EUR/SEK/HUF	5.60	0.05	5.50	5.70
47	Risk seeking	Cost	50 x population EUR/SEK/HUF	5.62	0.05	5.52	5.72
48	Risk seeking	Cost	10 x population EUR/SEK/HUF	5.67	0.05	5.57	5.77

Table SI23: Risk preference subgroup analysis: AMCEs for vaccine choice

	Subgroup	Attribute	Level	Estimate	SE	Lower CI	Upper CI
1	Risk averse	Side effects	1 in 10 000	0.00			
2	Risk averse	Side effects	1 in 100 000	0.27	0.04	0.20	0.34
3	Risk averse	Side effects	1 in 1 000 000	0.41	0.04	0.34	0.49
4	Risk averse	Effective	55%	0.00			
5	Risk averse	Effective	75%	0.50	0.04	0.42	0.57
6	Risk averse	Effective	95%	0.92	0.04	0.84	1.00
7	Risk averse	Country	China	0.00			
8	Risk averse	Country	Russia	0.16	0.05	0.07	0.25
9	Risk averse	Country	Great Britain	0.66	0.05	0.57	0.76
10	Risk averse	Country	USA	0.74	0.05	0.64	0.84
11	Risk averse	Country	Germany	0.92	0.05	0.82	1.02
12	Risk averse	Type	Live Virus Vaccine	0.00			
13	Risk averse	Type	Viral Vector Vaccine	0.06	0.04	-0.02	0.14
14	Risk averse	Type	Subunit Vaccine	0.07	0.04	-0.02	0.15
15	Risk averse	Type	mRNA Vaccine	0.23	0.04	0.14	0.31
16	Risk averse	People	1 million	0.00			
17	Risk averse	People	10 million	0.08	0.04	0.01	0.15
18	Risk averse	People	100 million	0.20	0.04	0.13	0.27
19	Risk averse	Duration	In 9 months	0.00			
20	Risk averse	Duration	In 6 months	0.04	0.04	-0.03	0.10
21	Risk averse	Duration	In 3 months	0.07	0.04	-0.00	0.14
22	Risk averse	Cost	100 x population EUR/SEK/HUF	0.00			
23	Risk averse	Cost	50 x population EUR/SEK/HUF	0.01	0.03	-0.06	0.07
24	Risk averse	Cost	10 x population EUR/SEK/HUF	0.07	0.04	-0.00	0.13
25	Risk seeking	Side effects	1 in 10 000	0.00			
26	Risk seeking	Side effects	1 in 100 000	0.14	0.04	0.07	0.21
27	Risk seeking	Side effects	1 in 1 000 000	0.27	0.04	0.19	0.34
28	Risk seeking	Effective	55%	0.00			
29	Risk seeking	Effective	75%	0.47	0.04	0.39	0.54
30	Risk seeking	Effective	95%	0.88	0.04	0.80	0.97
31	Risk seeking	Country	China	0.00			
32	Risk seeking	Country	Russia	0.18	0.05	0.09	0.28
33	Risk seeking	Country	Great Britain	0.71	0.05	0.61	0.82
34	Risk seeking	Country	USA	0.68	0.05	0.57	0.78
35	Risk seeking	Country	Germany	0.84	0.06	0.73	0.95
36	Risk seeking	Type	Live Virus Vaccine	0.00			
37	Risk seeking	Type	Viral Vector Vaccine	0.06	0.04	-0.03	0.14
38	Risk seeking	Type	Subunit Vaccine	0.01	0.04	-0.07	0.09
39	Risk seeking	Type	mRNA Vaccine	0.14	0.04	0.05	0.23
40	Risk seeking	People	1 million	0.00			
41	Risk seeking	People	10 million	0.09	0.04	0.02	0.16
42	Risk seeking	People	100 million	0.19	0.04	0.12	0.27
43	Risk seeking	Duration	In 9 months	0.00			
44	Risk seeking	Duration	In 6 months	0.12	0.04	0.04	0.19
45	Risk seeking	Duration	In 3 months	0.11	0.04	0.04	0.19
46	Risk seeking	Cost	100 x population EUR/SEK/HUF	0.00			
47	Risk seeking	Cost	50 x population EUR/SEK/HUF	0.03	0.04	-0.04	0.10
48	Risk seeking	Cost	10 x population EUR/SEK/HUF	0.07	0.04	0.00	0.14

Figure SI8: Screen shot of a vaccine decision scenario (translated into english)

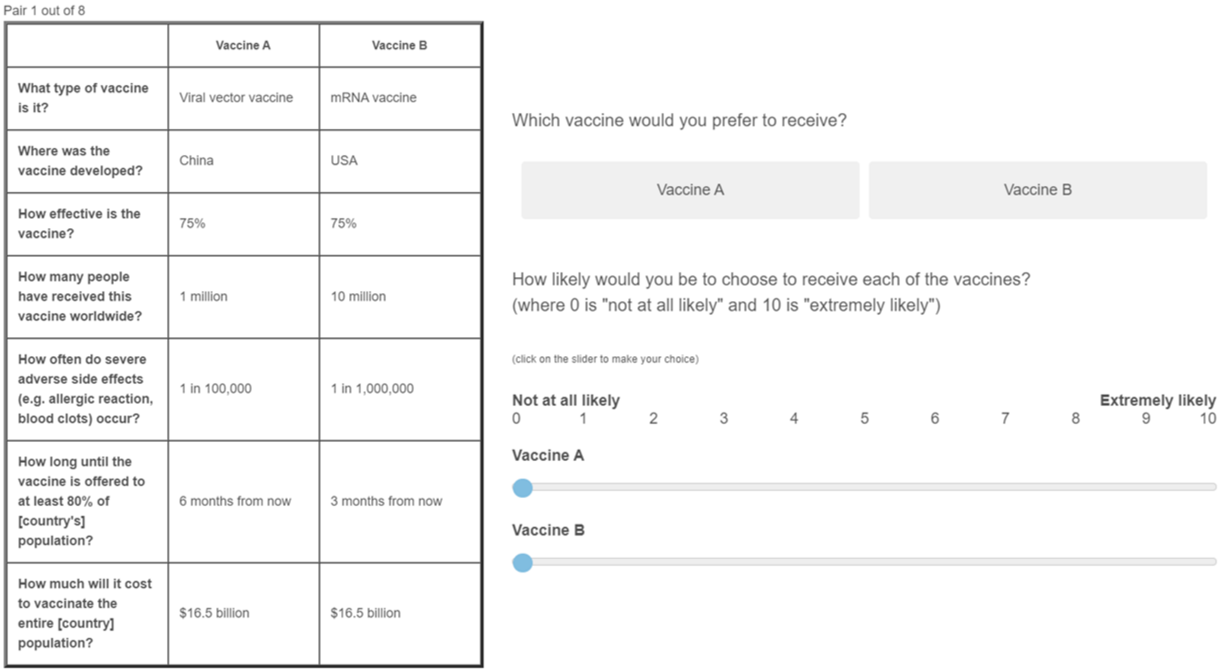

Pair 1 out of 8

	Vaccine A	Vaccine B
What type of vaccine is it?	Viral vector vaccine	mRNA vaccine
Where was the vaccine developed?	China	USA
How effective is the vaccine?	75%	75%
How many people have received this vaccine worldwide?	1 million	10 million
How often do severe adverse side effects (e.g. allergic reaction, blood clots) occur?	1 in 100,000	1 in 1,000,000
How long until the vaccine is offered to at least 80% of [country's] population?	6 months from now	3 months from now
How much will it cost to vaccinate the entire [country] population?	$16.5 billion	$16.5 billion

Which vaccine would you prefer to receive?

Vaccine A Vaccine B

How likely would you be to choose to receive each of the vaccines?
(where 0 is "not at all likely" and 10 is "extremely likely")

(click on the slider to make your choice)

Not at all likely Extremely likely
0 1 2 3 4 5 6 7 8 9 10

Vaccine A

Vaccine B

Note. Before being exposed to eight vaccine decision scenarios, respondents were exposed to (1) general information about vaccines, and (2) descriptions of the different vaccine types:

General information: Vaccines teach your immune system how to create antibodies that protect you from diseases. It's much safer for your immune system to learn this through vaccination than by catching the diseases and then treating them. Once your immune system knows how to fight a disease, it can often protect you for many years. Coronavirus vaccines that protect against Covid-19 do this by teaching your body to recognize a specific protein so your immune system can produce antibodies to protect you. In the case of the coronavirus, the vaccines teach your body to recognize and fight what is called the "spike protein" because of how it looks under a microscope. While all vaccines teach the body to recognize the "spike protein" and produce antibodies, vaccines can do this in different ways.

mRNA vaccines: mRNA vaccines introduce into the body genetic instructions called 'messenger RNA' that tells your body to produce the spike protein, but not other parts of the virus.

Subunit vaccines: Subunit vaccines introduce into the body fragments of the virus that include the spike protein, but not other parts of the virus.

Viral vector vaccines: Viral vector vaccines introduce into the body a harmless virus (the vector) that has had a gene added to it that tells the body to produce the spike protein, but not other parts of the virus.

Live virus vaccines: Live virus vaccines introduce into the body a weaker form of the virus. This weakened form would include the spike protein, but not be able to cause the disease.

Table SI24: Distribution of attribute levels for France

Attribute	n	Sample%
Side effects	28,532	
1 in 1,000,000	9,579	33.6%
1 in 100,000	9,536	33.4%
1 in 10,000	9,417	33.0%
Effectiveness	28,532	
95%	9,360	32.8%
75%	9,504	33.3%
55%	9,668	33.9%
Country	28,532	
Germany	5,607	19.7%
USA	5,769	20.2%
UK	5,670	19.9%
Russia	5,646	19.8%
China	5,840	20.5%
Vaccine type	28,532	
mRNA vaccine	7,140	25.0%
Subunit vaccine	7,042	24.7%
Viral vector vaccine	7,239	25.4%
Live virus vaccine	7,111	24.9%
Vaccined people	28,532	
100 millions	9,552	33.5%
10 millions	9,385	32.9%
1 million	9,595	33.6%
Vaccination coverage	28,532	
In 3 months	9,641	33.8%
In 6 months	9,353	32.8%
In 9 months	9,538	33.4%
Costs	28,532	
10x population EUR	9,511	33.3%
50x population EUR	9,571	33.5%
100x population EUR	9,450	33.1%

Table SI25: Distribution of attribute levels for Germany

Attribute	n	Sample%
Side effects	28,518	
1 in 1,000,000	9,448	33.1%
1 in 100,000	9,557	33.5%
1 in 10,000	9,513	33.4%
Effectiveness	28,518	
95%	9,485	32.3%
75%	9,525	33.4%
55%	9,485	33.3%
Country	28,518	
Germany	5,689	19.9%
USA	5,832	20.5%
UK	5,750	20.2%
Russia	5,721	20.1%
China	5,526	19.4%
Vaccine type	28,518	
mRNA vaccine	7,176	25.2%
Subunit vaccine	7,099	24.9%
Viral vector vaccine	7,169	25.1%
Live virus vaccine	7,074	24.8%
Vaccined people	28,518	
100 millions	9,487	33.3%
10 millions	9,471	32.2%
1 million	9,560	33.5%
Vaccination coverage	28,518	
In 3 months	9,520	33.4%
In 6 months	9,425	33.0%
In 9 months	9,573	33.6%
Costs	28,518	
10x population EUR	9,592	33.0%
50x population EUR	9,524	33.4%
100x population EUR	9,402	33.0%

Table SI26: Distribution of attribute levels for Sweden

Attribute	n	Sample%
Side effects	28,902	
1 in 1,000,000	9,638	33.3%
1 in 100,000	9,728	33.7%
1 in 10,000	9,536	33.0%
Effectiveness	28,902	
95%	9,558	33.1%
75%	9,822	34.0%
55%	9,522	32.9%
Country	28,902	
Germany	5,687	19.7%
USA	5,713	19.8%
UK	5,767	20.0%
Russia	5,853	20.3%
China	5,882	20.4%
Vaccine type	28,902	
mRNA vaccine	7,217	25.0%
Subunit vaccine	7,331	25.4%
Viral vector vaccine	7,167	24.8%
Live virus vaccine	7,187	24.9%
Vaccined people	28,902	
100 millions	9,629	33.3%
10 millions	9,723	33.6%
1 million	9,550	33.0%
Vaccination coverage	28,902	
In 3 months	9,642	33.4%
In 6 months	9,609	33.2%
In 9 months	9,651	33.4%
Costs	28,902	
10x population SEK	9,731	33.7%
50x population SEK	9,483	32.8%
100x population SEK	9,688	33.5%

Questionnaire

Gender

Now we would like to ask you a few questions about yourself. Please remember that your answers will remain confidential. What is your gender?

Male (0); Female (1)

Age

How old are you?

18 - 24 (0); 25 - 34 (0); 35 - 44 (1); 45 - 54 (1); 55 - 64 (2); 64 - 75 (2); 76 or older (2)

Education

What is the highest level of education you have successfully completed?

France

- Enseignement secondaire (sans obtenir de diplome) (0)
- Diplome de l'enseignement secondaire (baccalaureat ou equivalent) (1)
- Etudes superieures techniques/technologiques (sans obtenir de diplome) (1)
- Diplome d'etudes superieures de premier cycle (Bac +3) (2)
- Diplome d'etudes superieures techniques/technologiques (BTS, DUT ou equivalent) (2)
- Dipome d'etudes superieures de deuxieme ou troisieme cycle (Bac +5 : master, diplome d'ingenieur ou equivalent) (2)
- Doctorat, postdoctorat ou equivalent (Bac +8) (2)
- Prefere ne pas repondre (NA)

Germany

- Abgeschlossene Sekundarschulbildung/Ausbildung nicht abgeschlossen/Keine Antwort (0)
- Abschluss einer hoheren weiterführenden Schulbildung/Abschluss von beruflichem Fachgymnasium/Abgeschlossene Berufs- oder Fachausbildung (1)
- Abgeschlossene Universitatsausbildung (2)

Sweden

- Grundskoleutbildning (0)
- Gymnasie-, komvux-, folkhogskole-, yrkesforberedande eller larlingsutbildning (1)
- Kvalificerad yrkesutbildning (KY) eller annan yrkesutbildning (1)
- Akademisk utbildning grundniva: Hogskole- eller kandidatexamen (2)
- Akademisk utbildning avancerad niva: Magister- eller masterexamen (2)
- Akademisk utbildning forskarniva: Licentiats- eller doktorsexamen (2)

Coding: "Secondary education/education not completed/no answer" (0), "Higher secondary education/vocational specialized high school" (1), "University education"

Region

In which region of [country] do you currently reside?

France

Alsace (1)	Centre (7)	Languedoc-Roussillon (13)	Picardie (19)
Aquitaine (2)	Champagne-Ardenne (8)	Limousin (14)	Poitou-Charentes (20)
Auvergne (3)	Corse (9)	Lorraine (15)	Provence-Alpes-Cote d'Azur (21)
Basse-Normandie (4)	Franche-Comte (10)	Midi-Pyrenees (16)	Rhone-Alpes (22)
Bourgogne (5)	Haute-Normandie (11)	Nord-Pas-de-Calais (17)	
Bretagne (6)	Ile-de-France (12)	Pays de la Loire (18)	

Germany

Baden-Wurttemberg (1)	Bremen (5)	Niedersachsen (9)	Sachsen (13)
Bayern (2)	Hamburg (6)	Nordrhein-Westfalen (10)	Sachsen-Anhalt (14)
Berlin (3)	Hessen (7)	Rheinland-Pfalz (11)	Schleswig-Holstein (15)
Brandenburg (4)	Mecklenburg-Vorpommern (8)	Saarland (12)	Thuringen (16)

Sweden

Blekinge lan (1)	Jonkopings lan (7)	Skane lan (13)	Vasternorrlands lan (19)
Dalarnas lan (2)	Kalmar lan (8)	Sodermanlands lan (14)	Vastmanlands lan (20)
Gavleborgs lan (3)	Kronobergs lan (9)	Stockholms lan (15)	Vastra Gotalands lan (21)
Gotlands lan (4)	Norrbottens lan (10)	Uppsalas lan (16)	
Hallands lan (5)	Orebro lan (11)	Varmlands lan (17)	
Jamtlands lan (6)	Pstergotlands lan (12)	Vasterbottens lan (18)	

Vaccine hesitancy

Next we would like to ask you about your opinions regarding vaccinations. Please indicate whether you disagree or agree with the following statements.

- Getting vaccines is a good way to protect children from disease. (vax_att1)
- Generally, I do what my doctor recommends about vaccines. (vax_att2)
- New vaccines are recommended only if they are safe. (vax_att3)
- Children do not need vaccines for diseases that are not common anymore. (vax_att4)
- I am concerned about serious side effects of vaccines. (vax_att5)
- Some vaccines cause autism in healthy children. (vax_att6)
- Vaccinations are one of the most significant achievements in improving public health. (vax_att7)

Coding: We used a 5-Point scale from 'strongly disagree' (1) to 'strongly agree' (5).

Risk preferences

How do you see yourself: are you generally a person who is fully prepared to take risks or do you try to avoid taking risks? Please give a value between 0 (not at all willing to take risks) and 10 (very willing to take risks).

Attention checks

We integrated four attention checks throughout the whole survey.

- Two is greater than one. (check1)
- World War II came before World War I. (check2)
- Please select "neither agree nor disagree". (check3)
- Barack Obama was the first president of the United States of America. (check4)
