# Supplementary material for: Which vaccine attributes foster vaccine uptake? A cross-country conjoint experiment
Source: PLoS One. 2022 May 4;17(5):e0266003. doi: 10.1371/journal.pone.0266003 (PMC9067644; doi:10.1371/journal.pone.0266003)
Supplement: Attachment — Submitted filename: Response to Reviewers.docx [file pone.0266003.s002.docx]

*Exeter, January 2022*

**Response to Reviewers — PONE-D-21-35451**

Dear Prof. Daoust,

Thank you very much for the opportunity to revise and resubmit this manuscript. We greatly appreciate the feedback of the editor and the anonymous reviewers, which we believe has allowed us to significantly improve the manuscript.

In the current version, we have strengthened the literature review by adding some more recent research stemming from diverse countries (e.g., Japan, Philippines, Canada). Further, we have improved the methods section by providing more details, namely about methodological and statistical aspects that deviate from our pre-registration. Below, please find the detailed response to your and the reviewers’ individual remarks.

Thank you very much in advance for considering the revision of our manuscript. We are looking forward to hearing from you.

Sincerely,

Sabrina Stöckli, On behalf of the authors

**Comments to Academic Editor**

Thank you for evaluating our manuscript and providing detailed feedback.

*The decision to focus on three groups for the LCA comes a bit out of the blue without justification (line 242/243, 285/86, 359/60). Reading the pre-registered analysis plan, I see this vague mention to *five* groups: “(Based on other work by *author*, we anticipate using this scale to create a latent class model with five classes: strong vaccine supporter, supporter with reservations, vaccine hesitant, "anti-vax", and measurement error.)” I am puzzled. Can you please clarify/explain this gap (5 v/s 3 groups) and provide more information on, for example, the model fit indicators?*

1.1

Many thanks to the editor for raising this point of clarification. In our section “Pre-registration, deviations, and researcher degrees of freedom” we are now more transparent about our decision to conduct our analysis with three (instead of five) groups. In work now in press at *Human Vaccines & Immunotherpeutics*, two of the authors on this manuscript find that an LCA model using a nearly identical scale produces five interpretable classes that fits the data well in three (mostly) English speaking countries (US, UK, and Canada). Given that we had some basic expectations based on other data, we decided to include this in the preregistration. Yet, we also wanted to be transparent that our expectation was not borne out. The data we collected from the three non-English speaking countries featured in the current manuscript did not produce a five factor model that was easily interpretable and fit the data well. Instead, a three class model maximizes interpretability and fit. The focus of our research is not on the multidimensionality of vaccine attitudes, and hence it is more reasonable to use three instead of five subgroups for the vaccine hesitancy subgroup analysis. Opting for fewer groups makes the interpretation of our analysis not only simpler, but also clearer. Further, we now also provide more information on the model fit indicators, which underline that three classes is better (see lines 269–277, 321-329).

*On the deviation from the preregistered analysis plan. I would *not* require you to include Hungary, but of course the paper would benefit from including this sample. Would it be possible to include the sample at this stage?*

1.2

We thank the reviewer for this comment. If this is a “deal-breaker” for the editor or review, we are of course happy to include the Hungary data. That said, our preference is to keep them separate. First, there are subtle but important differences in the design and procedures. While the attributes and levels are substantially similar, other design elements are not. For instance, the Hungary study did not provide respondents with information about the different vaccine technologies. More trivially, the number of respondents and trials also differed. An important substantive difference of the Hungarian context is the set of vaccines available was larger than in other countries (vaccines from Russia and China were available, as were the “Western” vaccines.). In addition, analysis of the Hungary data uses variables not included in the France, Germany and Sweden data. Our concern is that clearly articulating the differences across the studies will do more to confuse the reader than the added value of similar results from another European country. We are not dogmatic on the point, and will follow guidance here. (Our goal is to publish the Hungary analysis separately where these different points of emphasis can be the focus rather than serve to confuse or distract the reader.)

Note that we are now more transparent and specific about our reasons to exclude Hungary from our manuscript (see lines 308–312).

*There might be a social desirability bias with the self-reported likelihood of vaccine uptake. Although you do not mention ‘social desirability bias’, this is what you probably refer to on lines 459-64. The discussion of this limitation seems, however, quite weak. I have a very partial view as I believe that there is much stronger evidence of a bias than not (Daoust et al. 2020, 2021; but see Larsen et al. 2020 for null results and Muzert and Selb 2020 for mixed findings) and I do not believe that claiming that the “vaccine intentions strongly related to actual vaccine behavior” is the best defence for the self-reported measure. First, I would tend to think that you should acknowledge that there is at least a substantial risk that the means are inflated by a social desirability bias. Second, and this is key, I believe that your research design makes the bias less problematic because the social desirability bias is very likely homogenous across the different treatments — allowing you to compare the differences without systematic bias.*

1.3

We agree with the need to elaborate on potential social desirability bias issues. We have revised the discussion section to give this matter greater attention (see lines 531–539).

*We note that you have provided funding information that is currently declared in your Funding Statement. However, funding information should not appear in the Acknowledgments section or other areas of your manuscript. We will only publish funding information present in the Funding Statement section of the online submission form.*

*Please remove any funding-related text from the manuscript and let us know how you would like to update your Funding Statement.*

1.4

We removed funding-related text from our manuscript. Please include the following statement to the Funding Statement section:

“This project received funding from the European Research Council (ERC) under the European Union’s Horizon 2020 research and innovation programme (grant agreement No. 682758). The funders had no role in study design, data collection and analysis, decision to publish, or preparation of the manuscript.”

1.5

Following your suggestion, we also changed our file naming as you have suggested, added additional details regarding participant consent in the method section, and a reference to figure 3 (new figure 4) (see lines 184–185).

**Comments to Reviewer 1**

Thank you for evaluating our manuscript and providing detailed feedback.

*This paper's contribution is international comparison and generalizability with multiple survey sites (France, Germany, Sweden). However, the introduction discussed only findings in a few countries. The survey experiments on COVID-19 vaccine is rapidly increasing and reports finding in various countries (including. for instance, Malawi (Kao, et., al 2021) and Netherlands (Reeskens, et., al. 2021)). I then recommend that the authors update the literature review part, including recent results of survey experiments.*

2.1

Thank you for highlighting this additional literature -- research regarding COVID is indeed moving quickly, and we are grateful for the opportunity to include reference to this additional work. THanks you for bringing these to our attention! As suggested, we added Kao et al. (2021), and Reeskens et al. (2021). We also added some other recent papers such as Argote et al. (2021) for evidence from Latin America; Kawata and Nakabayashi (2021) for evidence from Japan; and Legaspi, Malay and Lim (2021) for evidence from the Philippines (see lines 49, 68-71).

**Comments to Reviewer 2**

Thank you for evaluating our manuscript and providing detailed feedback.

*Might be worth looking at a public report conducted by the Media Ecosystem Observatory in Canada. They conduct a conjoint experiment, much like Motta (2021) and the authors, that looks at sub-group effects in vaccine attributes in Canada. They find similar results: people who indicate the will take a COVID-19 vaccine are more responsive to these characteristics than those who report they will not or who are uncertain* [*https://www.mcgill.ca/maxbellschool/files/maxbellschool/meo_vaccine_hesistancy_1.pdf*](https://www.mcgill.ca/maxbellschool/files/maxbellschool/meo_vaccine_hesistancy_1.pdf)

3.1

Many thanks to the reviewer for this recommendation. We added discussion of this report to our manuscript (see e.g., lines 68–71).

*A couple other studies provide nice support for the authors’ findings using real-world vaccines. A manuscript by Merkley and Loewen (*[*https://osf.io/ng9qh/*](https://osf.io/ng9qh/)*) find that people are more likely to differentiate by COVID-19 vaccine brand – as in, reluctance to take AstraZeneca and Johnson & Johnson compared to Pfizer and Moderna – if they are supportive of vaccines, and another article by these authors (JAMA Network Open, 2021) shows people are persuaded to take less preferred vaccines (like AZ and JJ) by highlighting their capacity to prevent death*

3.2

We agree that these studies provide nice support for our findings and have added the references to the discussion section of our manuscript (see lines 555-560).

*Why France, Germany and Sweden? I’d like to see some justification of the case selection. It might also be useful to provide a brief rundown of the vaccine rollout differences across these cases in terms of timeline and vaccine availability. I encourage the authors to at least include the country-by-country MMs in the main text for readers. I think it’s worth highlighting the unique cross-national scope of this study that gets glossed over by only presenting pooled results. If the results are similar, that’s interesting!*

3.3

We agree with the reviewer that it is important to clarify our case selection strategy, and have highlighted the reasoning and inferential benefits of our case selection (see lines 188–196). We also provide some information on the vaccine rollout, and added country marginal means (MMs) to the main text (see lines 389–403). We think that providing this contextual and country-specific information improves our manuscript.

*I wonder about the choice of attributes for side effects and effectiveness. Do people have a common understanding of the former? Or are some people thinking “fever” while others are thinking VITT? I recognize other studies have taken a similar approach, but the comparative weakness of the effect for side effects may be due to this lack of precision (and possibly R’s numeracy). As we saw with the VITT fiasco, people really do seem to be responsive to side effect issues. Similarly with effectiveness there is no clarity for respondents if the meaning is “symptomatic and asymptomatic infection” or just symptomatic infection or hospitalization or death. The Merkley and Loewen (2021) piece suggests people are responsive to symptomatic infection prevention and death prevention characteristics. Respondents may not be entirely clear on what is meant by effectiveness. Obviously, the authors cannot re-run these experiments, but I think it’s worth pointing out these limitations and proposing new research unpacks the side effect and effectiveness characteristics.*

3.4

Many thanks to the reviewer for pointing this out. We did not provide a definition for side effects or effectiveness in the survey, and we did not ask what respondents’ understandings of “side effects” / “effectiveness” were. As you correctly point out, understandings of side effects and effectiveness might vary, which might, in turn, lead to different responses. In designing the study, we considered a number of different ways to try and present this information that either further explained these concepts, or would improve numerical understanding. We felt that all of our attempts were left wanting in some way. We chose the formulation we did because it reasonably approximates how these concepts are discussed in COVID news coverage (“the vaccine is xx% effective” or “1 in xxx,xxx of patients had a serious side effect”). In this regard, we think that our attributes and levels correspond to the real world information environment people are likely to have when evaluating vaccines. We fully agree that with the reviewer (and by extension Merkley and Loewen) that how these concepts are presented are likely to have important consequences that we are unable to observe. We now acknowledge this limitation in our discussion, cite Merkley and Loewen (2021), and emphasize the importance of considering these potential limitations in future research (see lines 526–530).

*I’d like to see a picture of the conjoint task in the main text*

3.5

We thank the reviewer for this excellent suggestion. We now provide a picture of the conjoint task in the main text (see Fig. 1).

*I’d like to see some more effort to highlight effect sizes. These differences we observe in the means, how sizable are they? Might be useful to express differences in standard deviations and compare them to the sizes that have been found in previous work*

3.6

We appreciate the reviewer’s suggestion to give more attention to our effect sizes. In line with your comment, we now detail in the results section using the AMCEs (see lines 380, 389-40, 424) and compare them to other work in the conclusion (see lines 499–501). Overall, we find that our effect sizes are comparable with what other researchers found.

*These findings, in addition to the works pointed to above, lead to some interesting questions that the authors only start to hint at in their discussion. What are the mechanisms behind these sub-group differences? And what exactly do we make of the responsiveness of vaccine supporters to these vaccine characteristics? Would we really see slippage for vaccines with effectiveness in the 50 percent range in the real world? Or do we only really observe this because respondents are aware there are other available vaccines with superior characteristics? I think it’s worth thinking about whether these sub-group effects would generalize to other areas with less vaccine choice.*

3.7

We agree with the reviewer that our discussion is not very specific regarding the psychological mechanisms and practical relevance behind our sub-group differences. As such, we now discuss our sub-group effects in more detail (see lines 510–525).
